# NanoCore: core-genome-based bacterial genomic surveillance and outbreak detection in healthcare facilities from Nanopore and Illumina data

**DOI:** 10.1128/msystems.01080-24

**Published:** 2024-10-07

**Authors:** Sebastian A. Fuchs, Lisanna Hülse, Teresa Tamayo, Susanne Kolbe-Busch, Klaus Pfeffer, Alexander T. Dilthey

**Affiliations:** 1 Institute of Medical Microbiology and Hospital Hygiene, Heinrich Heine University, Düsseldorf, Germany; London School of Hygiene & Tropical Medicine, London, United Kingdom

**Keywords:** microbial genomics, bacterial outbreak analysis, Nanopore sequencing, hybrid approaches, MLST, healthcare pathogen surveillance

## Abstract

Genomic surveillance enables the early detection of pathogen transmission in healthcare facilities and contributes to the reduction of substantial patient harm. Fast turnaround times, flexible multiplexing, and low capital requirements make Nanopore sequencing well suited for genomic surveillance purposes; the analysis of Nanopore data, however, can be challenging. We present NanoCore, a user-friendly method for Nanopore-based genomic surveillance in healthcare facilities, enabling the calculation and visualization of cgMLST-like (core-genome multilocus sequence typing) sample distances directly from unassembled Nanopore reads. NanoCore implements a mapping, variant calling, and multilevel filtering strategy and also supports the analysis of Illumina data. We validated NanoCore on two 24-isolate data sets of methicillin-resistant *Staphylococcus aureus* (MRSA) and vancomycin-resistant *Enterococcus faecium* (VRE). In the Nanopore-only mode, NanoCore-based pairwise distances between closely related isolates were near-identical to Illumina-based SeqSphere^+^ distances, a gold standard commercial method (average differences of 0.75 and 0.81 alleles for MRSA and VRE; sd = 0.98 and 1.00), and gave an identical clustering into closely related and non-closely related isolates. In the “hybrid” mode, in which only Nanopore data are used for some isolates and only Illumina data for others, increased average pairwise isolate distance differences were observed (average differences of 3.44 and 1.95 for MRSA and VRE, respectively; sd = 2.76 and 1.34), while clustering results remained identical. NanoCore is computationally efficient (<15 hours of wall time for the analysis of a 24-isolate data set on a workstation), available as free software, and supports installation via conda. In conclusion, NanoCore enables the effective use of the Nanopore technology for bacterial pathogen surveillance in healthcare facilities.

**IMPORTANCE:**

Genomic surveillance involves sequencing the genomes and measuring the relatedness of bacteria from different patients or locations in the same healthcare facility, enabling an improved understanding of pathogen transmission pathways and the detection of “silent” outbreaks that would otherwise go undetected. It has become an indispensable tool for the detection and prevention of healthcare-associated infections and is routinely applied by many healthcare institutions. The earlier an outbreak or transmission chain is detected, the better; in this context, the Oxford Nanopore sequencing technology has important potential advantages over traditionally used short-read sequencing technologies, because it supports “real-time” data generation and the cost-effective “on demand” sequencing of small numbers of bacterial isolates. The analysis of Nanopore sequencing data, however, can be challenging. We present NanoCore, a user-friendly software for genomic surveillance that works directly based on Nanopore sequencing reads in FASTQ format, and demonstrate that its accuracy is equivalent to traditional gold standard short read-based analyses.

## INTRODUCTION

Genomic pathogen surveillance has become an essential tool for the detection, characterization, and prevention of healthcare-associated infections (1, 2) and for improved infection control (3–5). Genomic surveillance can be applied retrospectively to investigate epidemiologically indicated potential outbreaks or prospectively as part of “sequence first” regimes (6), involving the routine sequencing of indicator organisms of nosocomial importance (i.e., those spreading quickly and exhibiting multidrug resistance and/or virulence factors) and enabling the detection of cryptic transmissions and silent outbreaks. Key factors for the successful implementation of genomic surveillance include linking epidemiological data to genomic analyses, the speed at which sequencing data are generated and analyzed (7, 8), and the accuracy of calculated genetic distances between samples.

While most sequencing for genomic pathogen surveillance purposes in healthcare facilities has traditionally relied on the Illumina technology (9), the Oxford Nanopore technology (10) has become increasingly attractive. Advantages of Nanopore sequencing include rapid turnaround times, “real-time” data generation and output, the ability to sequence long fragments of DNA, and low capital costs; for healthcare facility pathogen surveillance, these may translate into reduced outbreak investigation times or the ability to implement genomic surveillance in resource-limited settings. In addition, throughput and error rates, previously limitations of the Nanopore technology (11, 12), have improved rapidly (13, 14), and Nanopore sequencing is widely used for the assembly of bacterial genomes (13, 15). During the COVID-19 pandemic, tens of thousands of viral genomes were sequenced with the Nanopore technology (16–18), demonstrating the potential of the technology for large-scale surveillance.

Challenges for the introduction of Nanopore sequencing in the healthcare pathogen surveillance context, however, include (i) the sensitivity of important established bacterial strain typing methods, such as multilocus sequence typing (MLST) (19, 20), core-genome MLST (cgMLST), or core-genome single-nucleotide polymorphism (cgSNP) (21), to sequencing errors, which may, despite recent progress, remain a concern for Nanopore sequencing data, and (ii) the potential requirement that newly generated isolate sequencing data should remain comparable to that of existing, typically Illumina-based, isolate sequencing data, for example, to enable the detection of low-intensity unrecognized outbreaks that may span several years.

Multiple studies on the use of Nanopore sequencing for the determination of bacterial sequence types and bacterial genomic epidemiology have shown encouraging results (22, 23). Larger-scale studies include Oh et al. (24), who reported mostly consistent, but non-identical, results between Nanopore- and Illumina-based analyses of 23 isolates of vancomycin-resistant *Enterococcus* (VRE); Hall et al. (25), who reported largely consistent results between Nanopore and Illumina for *Mycobacterium tuberculosis*; Liao et al. (26) and Liou et al. (27), who presented a Nanopore-based MLST typing approach for *Staphylococcus aureus*; Ferreira et al. (28), who demonstrated Nanopore-based sequence typing and phylogenetic analysis of methicillin-resistant *Staphylococcus aureus* (MRSA), obtaining results generally consistent with an Illumina-based analysis; and a number of studies on the successful application of Nanopore sequencing to sequence typing in *Salmonella* (29–32). Xian et al. (29), in particular, presented a homopolymer error reduction approach and explicitly considered the case of combining Illumina and Nanopore data in the same analysis. These results are complemented by a number of smaller-scale studies: Linde et al. (33) found consistent results between Illumina and Nanopore sequencing for two out of three evaluated species of highly pathogenic bacteria, represented by two isolates each; Greig et al. (34) compared the two technologies on two isolates of *Escherichia coli* and obtained largely concordant results; Tarumoto et al. (35) found that Illumina- and Nanopore-based sequence of two VRE isolates produced concordant results; Both et al. (36) applied Nanopore sequencing to improve the resolution of hospital VRE isolates; and Cao et al. (37) reported successful strain typing for three *Klebsiella pneumoniae* isolates.

With the exception of nanoMLST (26, 27), however, no tools have been presented for the user-friendly, integrated analysis of putative bacterial outbreaks directly from Oxford Nanopore sequencing reads in FASTQ format. NanoMLST was designed for the analysis of multiplex PCR data and implements a classical seven-gene MLST scheme, the resolution of which is often not sufficient for the fine-scale analysis of bacterial transmission chains (38). In addition, the important “hybrid” use case, in which only Nanopore data are used for some isolates and only Illumina data for others and which enables, for example, the fast investigation of urgent cases with Nanopore sequencing against a background of Illumina-sequenced other isolates, was only considered in Hall et al. (25) and Xian et al. (29).

Here, we present NanoCore, a user-friendly tool developed specifically to enable the effective use of the Oxford Nanopore technology for the genomic surveillance of bacteria and outbreak detection in healthcare facilities. NanoCore works directly based on unassembled Nanopore sequencing reads in FASTQ format, while also supporting the analysis of Illumina-sequenced isolates. We demonstrate the accuracy of NanoCore on two data sets of MRSA and VRE, comprising two species that are highly relevant in the hospital infection control and genomic epidemiology context (39, 40) and which exhibit a medium (MRSA) as well as high (VRE) degree of genome plasticity (41, 42). For validation, we compared NanoCore against Illumina-based analyses of the same samples with Ridom SeqSphere^+^ (43), a commercial “gold standard” software used by many hospital hygiene and infection control departments.

## RESULTS

### Overview of NanoCore

**Fig 1:**
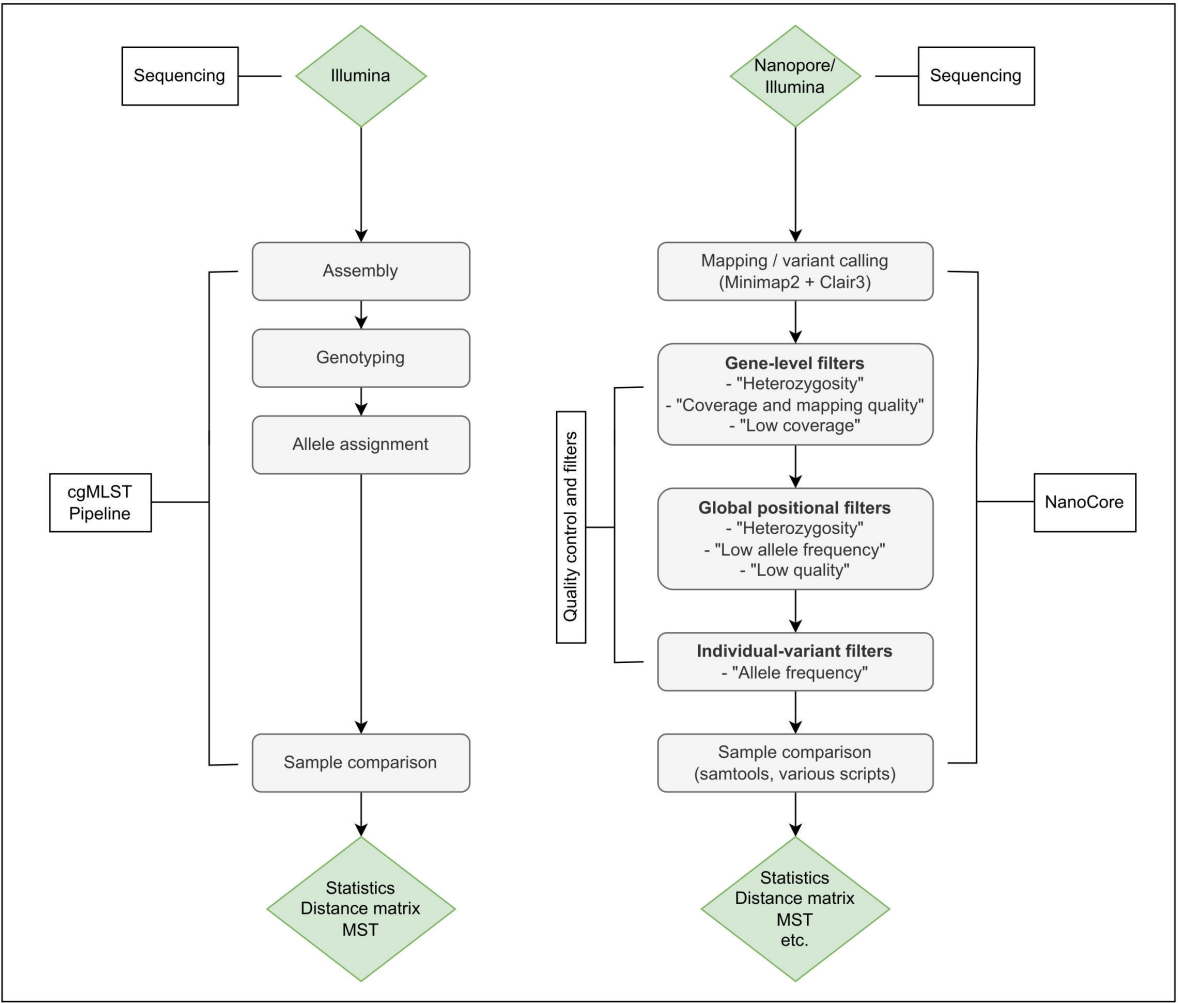
Overview of the NanoCore method (right), in comparison to a well-established method for the computation of cgMLST distances [SeqSphere^+^ (43), left]. In NanoCore, input reads are mapped to species-specific core genome references, followed by variant calling, the application of a tailored multilevel filtering strategy (“quality control and filters”), and the visualization of the analyzed sample using a minimum spanning tree (MST). Multilevel filtering removes false-positive variant calls; see Materials and Methods for details. Briefly, gene-level filters are applied at a per-isolate level and exclude genes affected by low mapping qualities, copy number, or structural variation; global positional filters affect all pairwise isolate comparisons and remove positions (i.e., one-base intervals along the reference genome) affected by short-range coverage fluctuations, base contexts challenging for Nanopore-based variant calling, and other technical artifacts of the variant calling process; finally, individual-variant filters remove false-positive variant calls in individual isolate pair comparisons.

NanoCore enables the investigation of putative bacterial outbreaks from Nanopore sequencing data, while also supporting the integrated analysis of Illumina-sequenced isolates (Fig. 1).

In NanoCore, input reads are mapped to a species-specific core genome reference, followed by variant calling, the calculation of pairwise isolate distances, and the visualization of the analyzed sample using a minimum spanning tree (MST). The robust computation of isolate distances from Nanopore data alone as well as in the “hybrid” analysis mode is enabled by a tailored multilevel filtering strategy, accounting for, e.g., copy number variation in the utilized core genome reference in individual isolates.

The pairwise isolate distance metric employed by NanoCore is similar, but not identical, to cgMLST: isolate distances in NanoCore are based on the number of species-specific core genome genes for which a difference in allelic state can confidently be asserted; however, no attempt is made to assign a fixed allele identifier to each analyzed gene in each isolate.

NanoCore, which is implemented in R and Perl, is freely available from GitHub.

### Validation experiment 1: *S. aureus* in Nanopore-only mode

In the first experiment, we benchmarked the Nanopore-only analysis mode of NanoCore on MRSA, representing a species of key relevance in the hospital outbreak context. Briefly, we assembled a 24-isolate benchmark data set from the biobank of University Hospital Düsseldorf’s Institute of Medical Microbiology and Hospital Hygiene, consisting of isolates collected between April 2017 and February 2022 and comprising three clusters of closely related isolates as determined by cgMLST analysis before. Per-sample Nanopore sequencing data were generated in a single multiplexed MinION R10 flow cell run (see Materials and Methods), and coverages ranged from 74× to 246× with an average of 120× (Fig. S1). NanoCore was benchmarked against an Illumina-based analysis of the same isolates with SeqSphere^+^, with per-sample coverages ranging from 33× to 187× (average: 101×).

Pairwise isolate distances computed by NanoCore (Table S1) were based on an average number of 1,856 compared genes per isolate pair, out of 1,864 genes present in the utilized *S. aureus* core genome data set (44). The gene-level filters affecting the largest number of genes were the “coverage and mapping quality” and “low coverage” filters, leading to the exclusion of 66 and 29 genes over all isolates, respectively (see Table S2; Fig. S2). Furthermore, 629 genomic positions (i.e., one-base intervals along the reference genome) were globally excluded from all pairwise distance calculations (most often due to the global positional heterozygosity filter; Table S3), and an additional 1,537 genomic positions were removed from individual pairwise comparisons (identified by the individual-variant filter; Table S4). By comparison, SeqSphere-computed distances were based on an average number of 1,832 analyzed genes per isolate and on an average number of 1,799 analyzed genes per isolate pair (Tables S5 and S6).

**Fig 2:**
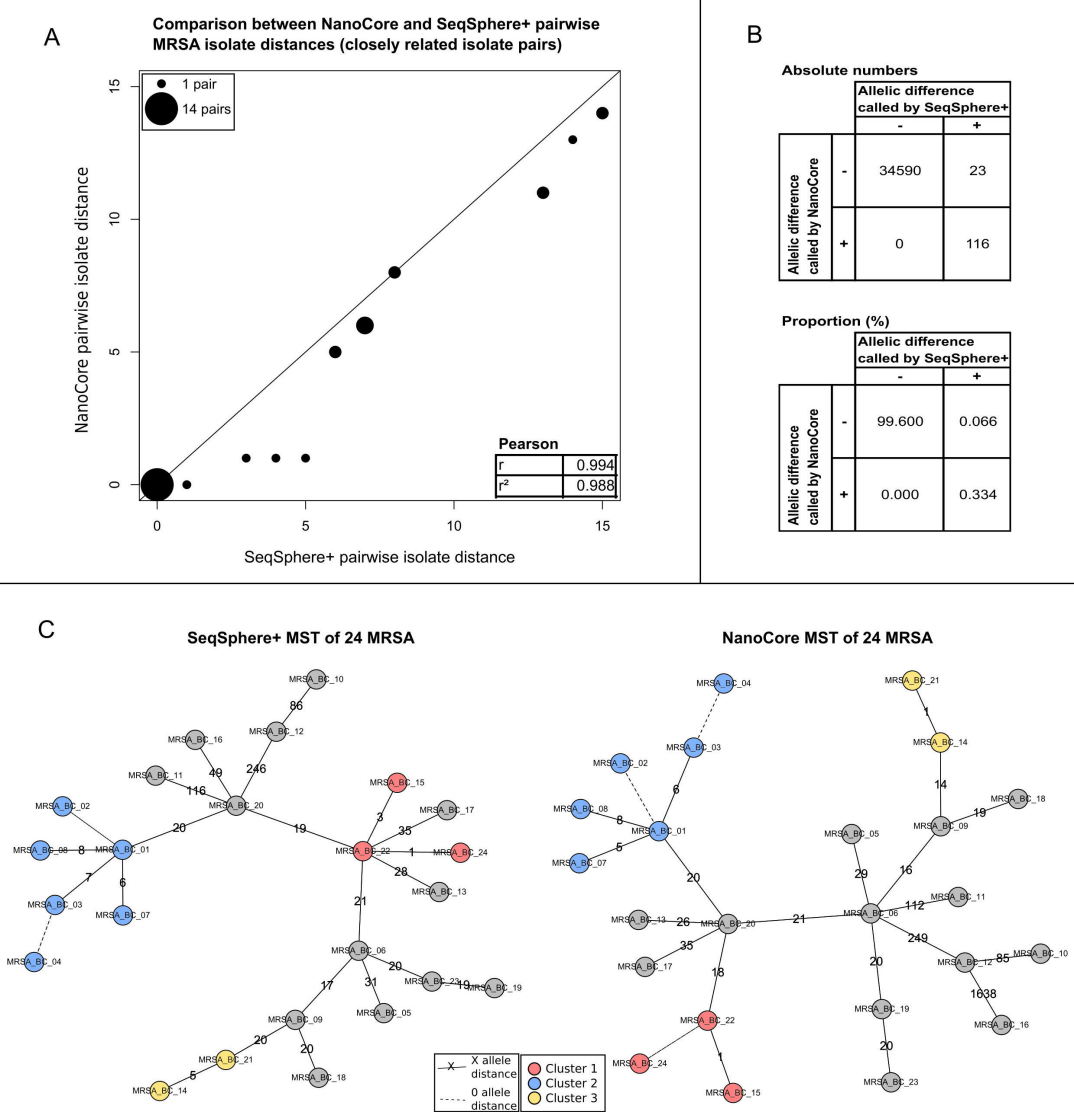
Analysis of 24 MRSA isolates. (**A**) Comparison of NanoCore- and SeqSphere^+^-based pairwise isolate distances for pairs of closely related isolates (SeqSphere^+^ distance ≤ 15), with Pearson correlation shown in the inset. Point sizes are scaled according to the number of pairwise distances with identical coordinates. (**B**) Comparison of individual-gene NanoCore and SeqSphere^+^ results across closely related isolate pairs (SeqSphere^+^ distance ≤ 15). Shown are results from genes that were analyzed by both NanoCore and SeqSphere^+^. (**C**) MSTs of the analyzed isolates based on SeqSphere^+^ (left) and NanoCore (right); clusters of closely related isolates, computed independently based on the output of SeqSphere^+^ and NanoCore, are shown as red, blue, and yellow circles. Dashed lines indicate a genetic distance of 0 alleles between the connected isolates; non-dashed lines are annotated with the specific genetic distance between the connected isolates.

NanoCore-computed pairwise distances (Table S1) were highly concordant with SeqSphere^+^ (Table S5; Pearson’s *r* = 1.000); for 47 out of 276 isolate pairs, the computed pairwise distances were identical. For the 19 pairs of closely related isolates with SeqSphere^+^ distances of ≤15 (i.e., covering the important use case of identifying pairs of isolates potentially related due to an infection chain context), NanoCore-computed pairwise distances were identical in four cases, and the average difference in pairwise distances was 0.75 (Fig. 2A).

We carried out an in-depth investigation of the observed differences between NanoCore and SeqSphere^+^ in the set of 19 pairs of closely related isolates with SeqSphere^+^ distances ≤ 15. First, we focused, across all included isolate pairs, on the 34,729 instances of pairwise gene comparisons present in both the NanoCore and SeqSphere^+^ analyses; of those, NanoCore and SeqSphere^+^ disagreed on only 23 instances (Fig. 2B). A manual investigation showed that the SeqSphere^+^ calls were likely correct in 8 of these 23 cases; 5 cases were classified as SeqSphere^+^ false-positive calls; and 10 cases remained ambiguous. The eight false-negative calls by NanoCore were exclusively due to the positions of the missed variants being close to the 5′ or 3′ends of a gene (Table S7); however, the detection of such variants is a known issue with the Clair3 variant caller used within NanoCore (GitHub issue: https://github.com/HKU-BAL/Clair3/issues/135; the proposed solution of padding the sequences of the included reference genes with “N” characters did not solve the problem). Next, we investigated the 15 out of 19 closely related isolate pairs for which a difference between the NanoCore- and SeqSphere^+^-computed distances was observed, independent of whether the gene pairs responsible for the observed differences were analyzed by both NanoCore and SeqSphere. In six cases, the observed differences in pairwise isolate distances could be attributed to a failure to detect true-positive allelic differences by NanoCore (usually driven by false-negative calls of variants close to the 5′ or 3′end of a gene) and in three cases to likely false-positive variant calls by SeqSphere^+^, and in six instances, the manual investigation showed that the distances calculated by neither approach were likely fully correct (see Table S8 for a full list of investigated pairwise differences).

Finally, clustering the isolates using a genetic distance threshold of 10 [consistent with recommendations by Schürch et al. (21)] produced the same sets of related isolates for NanoCore and SeqSphere^+^ (Fig. 2C), further demonstrating the high degree of concordance between NanoCore and SeqSphere^+^.

### Validation experiment 2: *Enterococcus faecium* in Nanopore-only mode

In the second experiment, we benchmarked the Nanopore-only mode of NanoCore on VRE, which may, due to a higher degree of genome plasticity, represent a challenge for the variant calling and filtering strategies employed by NanoCore. The selected 24 VRE isolates were taken from the biobank of University Hospital Düsseldorf’s Institute of Medical Microbiology and Hospital Hygiene, comprising two clusters of closely related isolates, and were collected between August and October 2021. Nanopore sequencing data were generated in two multiplexed MinION runs, and genome coverages ranged from 96× to 563× (mean: 273×; Fig. S3), compared to 51× to 108× (mean: 87×) for the Illumina data that were used for the comparative SeqSphere^+^ analysis.

In the case of VRE, we observed an increased number of genes removed by NanoCore’s default filters; pairwise distances (Table S9) were based on an average number of 1,397 compared genes, out of 1,423 genes present in the core genome (45). Consistent with an assumed effect of genome plasticity, the filter affecting the highest number of genes was the gene-level “heterozygosity” filter (607 genes removed in individual isolates; Table S2; Fig. S4), which is sensitive to variations in the genome structure. Furthermore, 877 genomic positions were globally excluded from all pairwise distance calculations (most often due to the global positional “heterozygosity” filter; Table S3), and 468 genomic positions were removed from individual pairwise comparisons (identified by the individual-variant filter; Table S4). By comparison, SeqSphere^+^-computed distances were based on an average number of 1,404 analyzed genes per isolate and on an average number of 1,385 analyzed genes per isolate pair (Tables S10 and S11).

**Fig 3:**
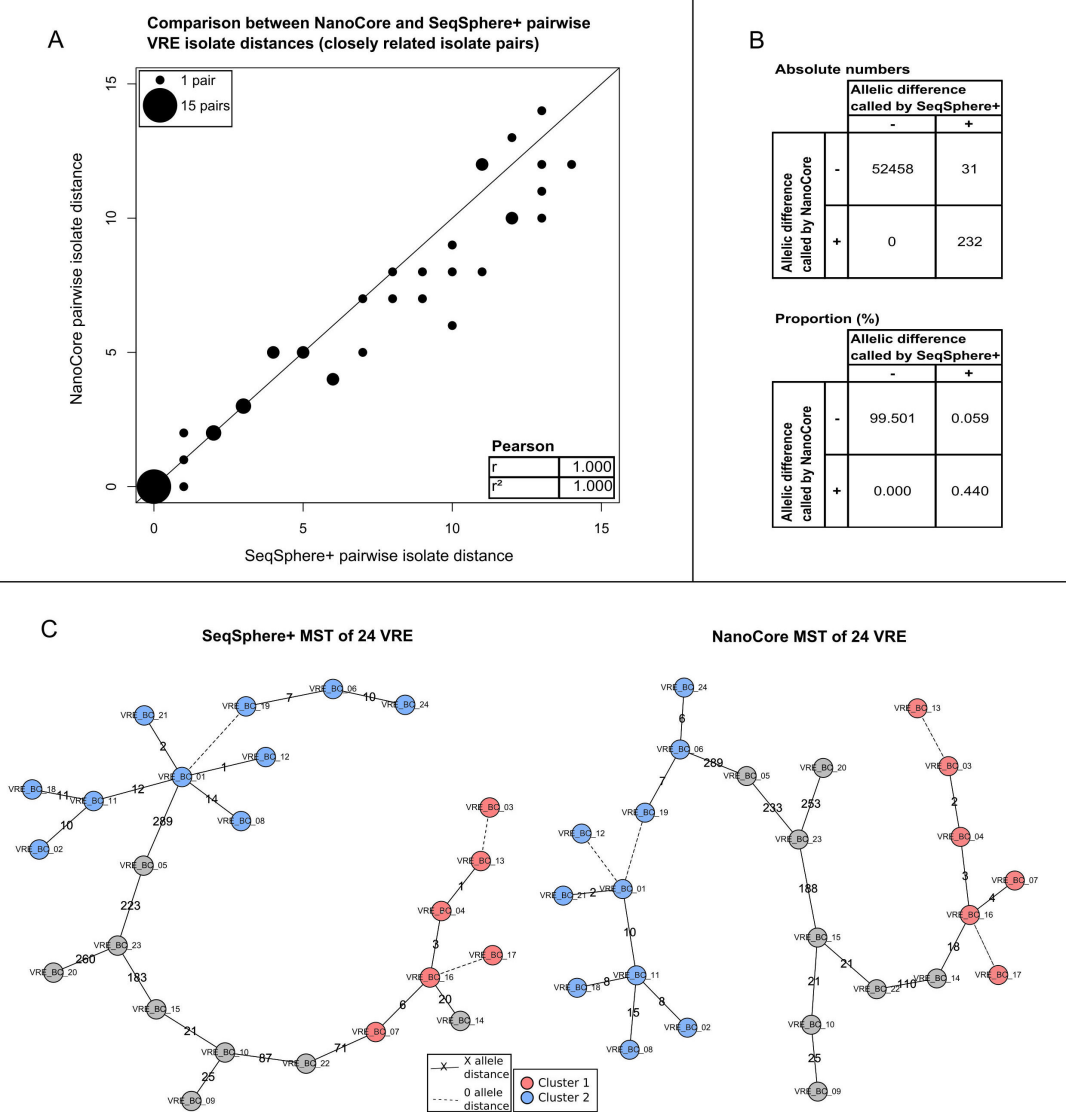
Analysis of 24 VRE isolates. (**A**) Comparison of NanoCore- and SeqSphere^+^-based pairwise isolate distances for pairs of closely related isolates (SeqSphere^+^ distance ≤ 15), with Pearson correlation shown in the inset. Point sizes are scaled according to the number of pairwise distances with identical coordinates. (**B**) Comparison of NanoCore- and SeqSphere^+^-based results on the level of individual genes across closely related isolates (SeqSphere^+^ distance ≤ 15). Shown are results from genes that were analyzed by both NanoCore and SeqSphere^+^. (**C**) Minimum spanning trees of the analyzed isolates based on SeqSphere^+^ (left) and NanoCore (right); clusters of closely related isolates, computed independently from the output of SeqSphere^+^ and NanoCore, are shown as red and blue circles. Dashed lines indicate a genetic distance of 0 alleles between the connected isolates; non-dashed lines are annotated with the specific genetic distance between the connected isolates.

As was the case for MRSA, the NanoCore-computed pairwise distances for VRE (Table S9) exhibited a high degree of concordance with SeqSphere^+^ (Table S10; Pearson’s *r* = 0.998 for all isolate pairs); for the 39 pairs of closely related isolates (SeqSphere^+^ distances ≤ 15), the degree of concordance for computed distances was higher (*r* = 1.000) and exhibited an average difference of 0.81 (Fig. 3A).

Furthermore, within the set of pairwise gene comparisons conducted by both SeqSphere^+^ and NanoCore in the set of closely related isolates, the two methods disagreed on only 31 out of 55,497 instances of pairwise gene comparisons (Fig. 3B), driven by differences in the allelic state called by SeqSphere^+^. Manual investigation showed that SeqSphere^+^ was likely correct in 24 of these 31 cases; five cases were classified as SeqSphere^+^ false-positive calls, and two cases remained ambiguous. False-negative calls by NanoCore were either due to low coverage (nine cases) or the positions of the missed variants being close to the 5′ or 3′ end of a gene (15 cases; Table S7). Finally, manual adjudication of the 25 out of 39 closely related isolate pairs for which a difference between the NanoCore- and SeqSphere^+^-computed distances was observed showed that 11 of these instances were due to false-negative calls by NanoCore (typically driven by exclusion of the variant-containing genes by the gene-level heterozygosity filter), 4 were due to likely false-positive calls by SeqSphere, and in 10 instances, the manual investigation showed that the distances calculated by neither approach were likely fully correct (Table S8).

Finally, isolate clusters computed using a genetic distance threshold of 15 [consistent with recommendations by Schürch et al. (21)] were identical between NanoCore and SeqSphere^+^ (Fig. 3C), demonstrating the high degree of consistency between the two methods.

### Validation experiment 3: evaluation of the “hybrid” mode of NanoCore on MRSA and VRE

To evaluate the “hybrid” analysis mode of NanoCore, we first assembled synthetic hybrid MRSA and VRE data sets for benchmarking purposes based on the sequencing data analyzed in the first two experiments; to assemble the hybrid data sets, the Nanopore and Illumina data from each isolate were not combined but treated as if they emanated from biologically different isolates, yielding two MRSA and VRE data sets with 48 isolates each.

We first evaluated the impact of NanoCore’s multilevel filtering strategy. For the “Nanopore” component of the hybrid data sets, gene-level filtering, which is applied to each isolate independently, produced the same results as in the first two experiments; for the “Illumina” component, gene-level filtering led to the exclusion of a median number of 374 and 79 genes for MRSA and VRE, respectively (Tables S12 and S13). The filters leading to the largest numbers of genes for the “Illumina” component were the gene-level “low coverage” filter (approximately 4,000 genes over all isolates in both data sets, Table S2) and the gene-level “coverage and mapping quality” filter, which had a particularly large effect in the MRSA data set (almost 6,500 genes over all isolates in both data sets; Table S2); correlations between the different filters are visualized in Fig. S5 and S6. Furthermore, 11,442 and 3,048 genomic positions were excluded by global positional filters for MRSA and VRE, respectively (Table S3), as well as 3,533 (MRSA) and 1,272 (VRE) positions from individual pairwise isolate distance calculations (identified by the individual-variant filter; Table S4).

**Fig 4:**
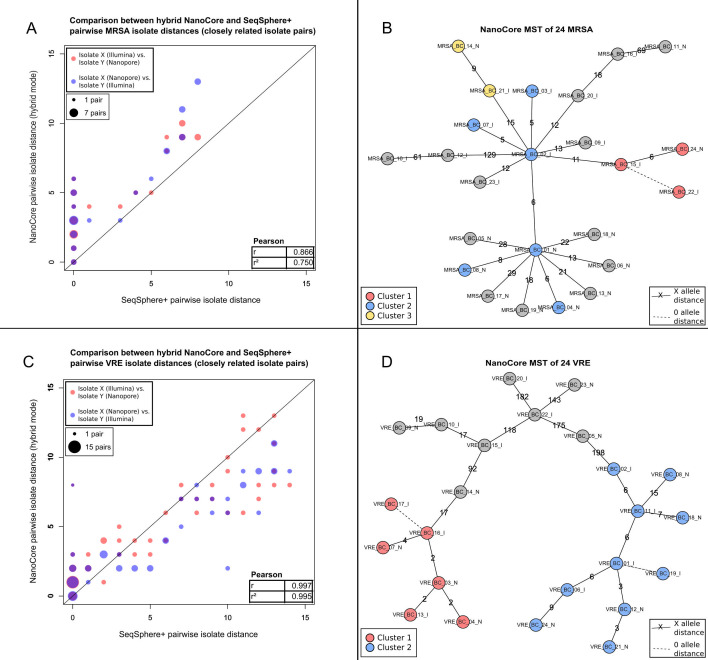
Evaluation of the “hybrid” mode of NanoCore on MRSA and VRE. (**A**) Comparison of “hybrid” NanoCore and SeqSphere^+^ pairwise isolate distances for pairs of closely related MRSA isolates (SeqSphere^+^ distance ≤ 15), with Pearson correlation shown in the inset. Point sizes are scaled according to the number of pairwise distances with identical coordinates. (**B**) NanoCore “hybrid” mode minimum spanning tree of the analyzed MRSA isolates, based on the first “hybrid” MRSA scenario (comprising 12 isolates for which only Nanopore data were used and 12 isolates for which only Illumina data were used; see Results); clusters of closely related isolates are shown as red, blue, and yellow circles. Dashed lines indicate a genetic distance of 0 alleles between the connected isolates; non-dashed lines are annotated with the specific genetic distance between the connected isolates. (**C**) Comparison of “hybrid” NanoCore- and SeqSphere^+^-based pairwise isolate distances for pairs of closely related VRE isolates (SeqSphere^+^ distance ≤ 15), with Pearson correlation shown in the inset. Point sizes are scaled according to the number of pairwise distances with identical coordinates. (**D**) NanoCore “hybrid” mode minimum spanning tree of the analyzed VRE isolates, based on the first “hybrid” VRE scenario (comprising 12 isolates for which only Nanopore data were used and 12 isolates for which only Illumina data were used; see Results); clusters of closely related isolates are shown as red and blue circles. Dashed lines indicate a genetic distance of 0 alleles between the connected isolates; non-dashed lines are annotated with the specific genetic distance between the connected isolates.

Within the two benchmarking data sets, we compared, for each pair of biological isolates (276 pairs in total per species), hybrid with single-technology pairwise isolate distances (Tables S14 and S15). Specifically, for two isolates *X* and *Y*, we compared distance_NanoCore_(*X*_Nanopore_,*Y*_Illumina_) and distance_NanoCore_(*X*_Illumina_,*Y*_Nanopore_) (the “hybrid” distances) with distance_NanoCore_(*X*_Nanopore_,*Y*_Nanopore_) and distance_SeqSphere_(*X*_Illumina_,*Y*_Illumina_) (the “single-technology” distances); the first subscript indicates the utilized pairwise distance computation method, and the subscripts of *X* and *Y* indicate the sequencing technology data type. We found that hybrid isolate distances were generally highly concordant with single-technology isolate distances; specifically, over 552 evaluated hybrid distances for each species, hybrid distances, and the Nanopore-based distances exhibited a correlation (Pearson’s *r*) of 1.000 (MRSA) and 0.985 (VRE); hybrid distances and Illumina-based SeqSphere^+^ distances exhibited a correlation of 0.981 (MRSA) and 0.985 (VRE). When considering only pairs of closely related isolates (SeqSphere^+^ distance ≤ 15), we observed an average difference between NanoCore- and SeqSphere^+^-based distances of 3.44 and a correlation of 0.866 for MRSA (19 isolate pairs; Fig. 4A; Table S14) and an average difference of 1.95 and a correlation of 0.997 for VRE (39 isolate pairs; Fig. 4C; Table S15).

Next, to investigate the accuracy of isolate clustering in the “hybrid” mode, we created three “hybrid” scenarios for both MRSA and VRE, which all comprised the full set of 24 biological isolates of the corresponding species and in which only Nanopore data were used for one randomly assigned half of the biological isolates and only Illumina data for the other half. Each “hybrid” scenario was analyzed as an independent NanoCore run, and clustering was carried out using the same distance thresholds as in the first two validation experiments. Within each “hybrid” scenario and for both species, we found perfect agreement between the computed clusters and the single-technology clustering results from the first two experiments (Fig. 4B and D).

To further characterize potential error modes of the “hybrid” analysis mode of NanoCore, we carried out an in-depth analysis of the first “hybrid” scenario for each species, focusing on 18 out of 19 (MRSA) and 30 out of 39 (VRE) closely related isolate pairs (SeqSphere^+^ distance ≤ 15) for which a difference between SeqSphere^+^ and NanoCore (in “hybrid” mode) distances was observed within the respective first “hybrid” scenario. For MRSA, 12 of the 18 differences were accounted for by “hybrid” distances; for VRE, 22 of 30. Further manual investigation showed that 5 of the 18 differences for MRSA were due to false-positive or false-negative calls by NanoCore, 2 were errors by SeqSphere^+^, and in 11 cases, neither distance was likely fully correct (Table S7). For VRE, 11 of the observed 30 discrepancies were likely driven by false-positive or false-negative calls by NanoCore; 2, by errors by SeqSphere^+^; and in 17 cases, neither distance was likely fully correct. Across both species, false calls by NanoCore were often due to the exclusion of the variant-containing genes by the gene-level “low coverage” filter, due to the corresponding variants being close to the 5′ or 3′ borders of a gene, or due to variant calling artifacts in low-coverage regions that were not removed by any of the coverage-related filters (Tables S7 and S8).

### Nanopore basecaller comparison

We also assessed the sensitivity of NanoCore to the specific Nanopore basecalling algorithm used. Briefly, we re-basecalled all generated Nanopore data with Dorado and carried out a comparison with Guppy, which all other results presented in this paper are based on (see Materials and Methods for details). First, we analyzed Dorado-based inter-sample genetic distances for MRSA and VRE (Tables S16 and S17) and found them to be highly similar to Guppy-based inter-sample genetic distances (Pearson’s *r* = 1.000 and 1.000 across all sample pairs for both MRSA and VRE; mean inter-sample genetic distance differences for closely related isolate pairs of 0 and 0.276 for MRSA and VRE, respectively; Tables S18 and S19). Second, we created synthetic “mixed” Dorado/Guppy analyses in which only Dorado data were used for one randomly assigned half of the isolates within each species and only Guppy data for the other half. Observed inter-sample genetic distances (Tables S16 and S17) in this experiment were highly similar to Guppy-only-based inter-sample genetic distances (Pearson’s *r* = 1.000 and 1.000 across all sample pairs for both MRSA and VRE; mean inter-sample genetic distance difference for closely related isolate pairs of 0 and 0.197 for MRSA and VRE, respectively; Tables S18 and S19), confirming that NanoCore results remain virtually identical when data generated using two different basecalling algorithms are combined in the same analysis.

### Computational performance

Analysis of the 24-isolate data sets described above with NanoCore (eight threads) took <15 hours of wall time and <5 Gb of RAM per experiment on an AMD Ryzen Threadripper 3970X system with 3.7 Ghz. Detailed runtime and computational requirement statistics are reported in Table S20.

## DISCUSSION

We have presented NanoCore, a user-friendly method for Nanopore-based genomic surveillance of bacteria and outbreak detection in healthcare facilities. NanoCore does not require any preprocessing of the Nanopore read data, accepting unassembled sequencing reads in FASTQ format as input. In addition to Nanopore sequencing, NanoCore also supports the analysis of Illumina-sequenced isolates. Important use cases of this include the selective application of Nanopore sequencing to urgent cases, leveraging the technology’s rapid data generation capabilities, as well as the complete transition of a hospital’s surveillance platform from Illumina to Nanopore sequencing without having to exclude or re-sequence older isolates for which only Illumina data are available.

We validated NanoCore on two independent 24-isolate data sets of MRSA and VRE, species highly relevant to the field of hospital hygiene and infection control. The validation experiments demonstrated identical clustering results between NanoCore in both evaluated modes (Nanopore-only and “hybrid”) and SeqSphere^+^, a commercial gold standard method, for both species. Pairwise isolate distances for closely related isolates based on NanoCore in the Nanopore-only mode were near-identical to those of SeqSphere^+^ (average differences of 0.75 for MRSA and 0.81 for VRE); for NanoCore in the “hybrid” mode, the average difference in pairwise isolate distances between NanoCore and SeqSphere^+^ was found to be increased (average differences of 3.44 and 1.95 for MRSA and VRE, respectively) but remained at a low level. Hospital outbreak investigations typically focus on distinguishing between related and non-related isolates and on the fine-scale structure of relatedness within the set of related isolates. By contrast, the determination of accurate pairwise isolate distances for more distantly related isolates can be relevant in the context of phylogenetics, but typically not in the context of outbreak investigations. The validation experiments, thus, demonstrated the near-equivalence between NanoCore and SeqSphere^+^ for the use case of bacterial genomic surveillance and outbreak detection in healthcare facilities.

NanoCore employs a multilevel filtering strategy to heuristically reduce the potential impact of false variant calls on computed pairwise sample distances. First, gene-level filters are applied at a per-isolate level to detect read mapping ambiguities as well as duplications or deletions of individual genes, which are associated with variant calling artifacts and which were occasionally observed in the analyzed isolates (Fig. S7), the classification of the analyzed genes as “core” notwithstanding. Consistent with the higher genomic plasticity of *E. faecium,* gene-level filters and the “heterozygosity” filter in particular had a substantially larger effect in VRE than in MRSA (Table S2). Second, positional filters capture technical artifacts of the variant calling process and base contexts that pose challenges for Nanopore-based variant calling, as well as drops in coverage. Positional filtering is implemented in a way that initially identifies potentially problematic positions on a per-sample basis, which are subsequently propagated across the complete data set (i.e., excluded from all distance calculations); this is based on the rationale that the properties that render individual positions challenging are typically shared between isolates, even if the heuristics employed to detect these positions are not activated in every individual isolate. Last, individual isolate-distinguishing variant calls are filtered based on the allele frequency of the called variant in the involved isolates; this step reduces the impact of false-negative variant calls. Because of the increased rate of homopolymer errors in Nanopore sequencing, INDEL calls are generally ignored by NanoCore; of note, Xian et al. similarly proposed a heuristic approach for homopolymer correction (29).

Our in-depth investigation of differences between NanoCore and SeqSphere^+^ for pairs of closely related isolate pairs showed that these were almost exclusively driven by false-negatives (i.e., NanoCore failing to detect a true isolate-distinguishing variant), which were often caused by a known variant calling issue of the Clair3 variant caller in the case of MRSA and often related to the gene-level “heterozygosity” filter in the case of VRE. Improvements to the Clair3 variant caller, or integration of another variant calling algorithm, may reduce these errors in the future. In the “hybrid” mode, we also observed false-positive calls by NanoCore (i.e., NanoCore erroneously calling an isolate-distinguishing variant that is not really present); these could be addressed by the integration of an Illumina-optimized variant calling approach (46) in future releases of NanoCore. In addition, the filtering strategy of NanoCore could be optimized for short-read data, for example, with respect to the increased coverage fluctuations (Fig. S6) and lower mapping qualities (Fig. S6) observed in short-read data; such potential for optimization was particularly apparent for the MRSA data set, in which increased coverage fluctuations in the short-read data led to the exclusion of a comparably high number of genes (Table S2), contributing to increased discrepancies between “hybrid” NanoCore and SeqSphere^+^ for this species. Importantly, while most observed differences between SeqSphere^+^ and NanoCore were due to NanoCore, we also observed false-positive calls by SeqSphere^+^ in all experiments.

We also investigated to which extent NanoCore results are influenced by the choice of Nanopore basecalling algorithm; specifically, we found that inter-sample genetic distances based on the most recent basecalling algorithm (Dorado) were near-identical to inter-sample genetic distances based on the previous generation of basecalling algorithms (Guppy) and that data produced by the two basecalling algorithms can be combined in the same analysis. New Nanopore sequencing data sets can, thus, be continuously integrated as they become available, without the need to re-basecall all existing data when a new basecaller becomes available.

NanoCore has a number of limitations. First, NanoCore requires a core genome reference; while these are available (https://www.cgmlst.org/ncs) for the large majority of clinically important species, there are still microbial species for which a core genome data set has not been defined yet. Second, by design, NanoCore will only detect isolate-distinguishing variants in the core genome; in some instances, whole-genome-based approaches also accounting for extrachromosomal genome information (i.e., from plasmids) may offer increased resolution for the fine-scale analysis of otherwise closely related isolates (38). Third, NanoCore does not assign a standardized allele identifier to the analyzed genes; NanoCore does, thus, not enable the comparison of isolates based on allele identifiers alone (47), which can be important, e.g., in the context of inter-institutional outbreak investigations in which the sharing of raw sequencing data is not possible. Fourth, in the current implementation, NanoCore may not scale to the analysis of very large data sets; in future releases, this could be addressed by limiting the computation of full pairwise distances to closely related isolates while relying on an approximate distance metric, e.g., based on Mash (48), otherwise. Fifth, NanoCore does not support the analysis of isolates based on *de novo* assembly. While limiting, as discussed above, the resolution of NanoCore to the core genome, the advantage of this approach is that NanoCore can also be applied to lower-coverage data sets. For example, we obtained virtually identical results for the MRSA data set after downsampling the Nanopore input data to 50% of its original size (data not shown); in addition to demonstrating robustness, this result indicates that NanoCore may also support Nanopore multiplexing schemes with more than 24 isolates per flow cell.

### Conclusion

NanoCore is a user-friendly method for genomic surveillance and outbreak detection in healthcare facilities based on the Oxford Nanopore sequencing technology. In two independent validation experiments based on MRSA and VRE, we demonstrated consistency between NanoCore and SeqSphere^+^, a gold standard commercial method. NanoCore also supports the analysis of Illumina-sequenced samples. In conclusion, NanoCore enables the effective use of the Nanopore technology for bacterial pathogen surveillance in healthcare facilities, the potential advantages of which include low capital costs and reduced sample-to-result turnaround times.

## MATERIALS AND METHODS

### Analyzed bacterial isolates and core genome references

Twenty-four VRE and 24 MRSA isolates were selected from the isolate collection of the Institute of Medical Microbiology and Hospital Hygiene of Düsseldorf University Hospital. All isolates had been previously sequenced with Illumina and analyzed with SeqSphere^+^ as part of the Institute’s routine surveillance activities; the analyzed isolates were selected to represent different degrees of genetic relatedness (see Results). For the generation of the Nanopore data, DNA was obtained from cryostocks of the selected isolates that were thawed and re-cultured.

For the analysis of these samples with NanoCore, we selected well-established core genome references for *Staphylococcus aureus*, comprising 1,864 core genes and 1.70 Mbp of sequence (44), as well as for *Enterococcus faecium*, comprising 1,423 core genes and 1.35 Mbp of sequence (45) .

### Bacterial culture and DNA extraction

Bacterial isolates were cultured employing routine overnight LB (lysogeny broth) culture protocols at 37°C. DNA was extracted using the Qiagen DNeasy UltraClean Microbial Kit according to the manufacturer’s instructions. DNA concentrations and quality were checked with NanoDrop, and 100 ng of DNA was diluted to fit the desired concentration of 5 ng/µL.

### Nanopore sequencing and demultiplexing

Nanopore sequencing was carried out on the Oxford Nanopore MinION device. DNA concentrations were measured using Qubit. Sequencing libraries for MRSA were prepared using the Oxford Nanopore ligation sequencing gDNA native barcoding kit SQK-NBD112-24 and sequenced on “FLO-MIN112” R10 flow cell, multiplexing 24 isolates per flow cell. Sequencing data for VRE were generated in two separate MinION runs, multiplexing 13 and 11 isolates per flow cell, based on the SQK-NBD112-24 kit with a “FLO-MIN112” R10 flow cell and based on the SQK-NBD114-24 kit with a “FLO-MIN114” R10.4 flow cell, respectively. Reads were basecalled and demultiplexed using Guppy (version 6.1.5) and Dorado (version 0.7.1). All presented Nanopore analyses apart from the Nanopore basecaller comparison were based on the Guppy-basecalled data. Per-isolate sequencing data statistics are shown in Table S21.

### Illumina sequencing and demultiplexing

Illumina sequencing data were generated for routine surveillance purposes and over multiple sequencing runs. DNA quality control was carried out using the Fragment Analyzer and NanoDrop instruments. Sequencing libraries were prepared using the Illumina Nextera XT DNA Library Preparation Kit “FC-131-1096” for 96 samples. Post-library-prep QC was carried out using the Fragment Analyzer and NanoDrop instruments as well as using Fluorometric Assay for concentration checks. Samples were prepared by equimolar pooling (including additional quality control) and sequenced with the MiSeq v2 500 cycle kit (251 - 8 - 8 – 251). Post-sequencing processing, quality control, and demultiplexing were carried out on the instrument. Per-isolate sequencing data statistics are shown in Table S21.

### NanoCore

NanoCore is based on the following key steps: (i) for each isolate, mapping of the generated sequencing reads using minimap2 (49) to a species-specific core genome reference (using flags „-x map-ont“ or “-x sr” depending on the type of sequencing reads); (ii) for each isolate, detection of variants in core genome genes using the Clair3 variant caller (50) (with flags „--include_all_ctgs“ and „-m /path/to/model“ set according to the type of input data); (iii) computation of pairwise sample distances (see below); (iv) generation of a MST, visualizing the genetic structure of analyzed isolates and various results and quality control tables.

NanoCore is implemented in Perl; the MST step is implemented in R (51). BAM files are manipulated using samtools (52). NanoCore is available under the MIT license and can be installed via conda.

Input sequencing data are specified using a simple sample sheet in tab-separated format; in addition, the user specifies a species-specific core genome reference file. Reference files for eight bacterial species (Table S22) are included in the NanoCore package. In addition, the user may specify a minimum coverage threshold (default 20) and the number of threads used for components of the pipeline that support multithreading.

The genetic distance between two isolates in NanoCore is computed based on the number of genes that confidently, i.e., after application of gene-level, positional, and individual-variant filters (see below), differ in the allelic state. Formally, for a pair of isolates *X* and *Y*, the set of candidate pair-distinguishing variants is defined as the set of non-shared variant calls from the Clair3-generated VCF files for *X* and *Y*, where a candidate variant is defined by its location (gene and position) and the called variant allele. The set of candidate variants is filtered by (i) removing all INDEL variants, (ii) removing all variants located in genes flagged by gene-level filters as suspicious in isolates *X* or *Y*, (iii) removing all variants at positions flagged by global positional filters, and (iv) removing all variants flagged by the individual-variant filter. The genetic distance between *X* and *Y* is then defined as the number of core genome genes for which one or more variants remain in the set of candidate variant pairs post-filtering. We note that the NanoCore approach to computing genetic distances is similar, but not identical, to cgMLST, as no attempt is made by NanoCore to explicitly determine and label with an allele identifier the allelic state of individual genes.

### Gene-level, positional, and individual-variant filters

Gene-level filtering is carried out independently for each isolate by NanoCore; the aim of gene-level filtering is to identify specific genes in individual isolates that exhibit an increased probability of unreliable variant calling results. Gene-level filters comprise (i) the gene-level “heterozygosity” filter, which marks genes in which more than 50% of Clair3 variant calls are heterozygous; (ii) the gene-level “coverage and mapping quality” filter, which flags genes that exhibit average per-read mapping qualities of <55 and average coverages that deviate by more than 25% from the average coverage of the isolate (both conditions need to be satisfied for this filter to be activated); and (iii) the gene-level “low coverage” filter, which marks genes in which more than 10% of positions exhibit a coverage below the minimum coverage threshold.

Global positional filters flag individual positions with potentially problematic variant calling results; these are ignored across the entire analyzed data set. Global positional filters comprise (i) the positional “heterozygosity” filter, which flags positions with a heterozygous call in at least one isolate; (ii) the positional “low allele frequency” filter, which marks variant positions at which the called variant allele has <50% allele frequency in the FASTQ sequencing reads in at least one isolate (determined using the “allele frequency” tag in the VCF produced by the variant caller); (iii) the positional “low quality” filter, which marks all positions at which a Clair3 variant call was annotated with the “LowQual” tag in at least one isolate; and (iv) the positional “low coverage” filter, which flags all positions with coverage below the specified minimum coverage in at least one isolate.

Last, the individual-variant filter is applied to all candidate variants potentially distinguishing two isolates *X* and *Y* remaining after the application of the other filters; the aim of the individual-variant filter is to remove false-positive pair-distinguishing variants that arise from false-negative variant calls in either *X* or *Y*. Let *a* be the variant allele of the candidate pair-distinguishing variant and assume without loss of generality that *a* was called in *X*, but not in *Y*; a variant passes the individual-variant filter if and only if the allele frequency of *a* in the FASTQ reads of *Y* is less than 20% (determined with the “mpileup” function of samtools).

### SeqSphere^+^ comparison

Illumina sequencing data were analyzed with Ridom SeqSphere^+^ (43) using default settings for the analyzed species; pairwise genetic isolate distances based on cgMLST and the sets of analyzed genes per isolate were extracted from SeqSphere^+^ default output using custom scripts. For the presented analyses, the cgMLST-based distance metric of SeqSphere^+^ was compared to the cgMLST-like distance metric of NanoCore.

### Manual adjudication of differences between SeqSphere^+^ and NanoCore

Manual adjudication of differences between SeqSphere^+^ and NanoCore was based on the visual inspection of the aligned Illumina and/or Nanopore sequencing reads using the Integrative Genomics Viewer tool (version 2.11.0) (53).

### Clustering of closely related isolates

For a given maximum genetic distance *d*, clusters of closely related isolates are defined as the connected components of the graph *G* = (*V*, *E*), where *V* are the analyzed isolates and an edge *e* connecting two isolates *X* and *Y* exists if and only if the pairwise genetic distance between *X* and *Y* is ≤*d*. For the analysis of the VRE isolates, *d* was set to 15; for the analysis of the MRSA isolates, *d* was set to 10, in line with recommendations by Schürch et al. (21).

## Data Availability

The utilized sequencing data are available under BioProject ID PRJNA1012291. NanoCore is available on GitHub (https://github.com/SebastianMeyer1989/NanoCore; DOI: https://doi.org/10.5281/zenodo.13269259) and licensed under the MIT license.

## SUPPLEMENTAL MATERIAL

Figure S1 msystems.01080-24-s0001.pdf Experiment 1 (MRSA): basic statistic plots.

Figure S2 msystems.01080-24-s0002.pdf Heatmaps of eye-catching and potentially due to different filters excluded genes in the Nanopore-only validation experiment 1 on MRSA data.

Figure S3 msystems.01080-24-s0003.pdf Experiment 2 (VRE): basic statistic plots.

Figure S4 msystems.01080-24-s0004.pdf Heatmaps of eye-catching and potentially due to different filters excluded genes in the Nanopore-only validation experiment 2 on VRE data.

Figure S5 msystems.01080-24-s0005.pdf Heatmaps of eye-catching and potentially due to different filters excluded genes in the hybrid validation experiment 3 part 1 on MRSA data.

Figure S6 msystems.01080-24-s0006.pdf Heatmaps of eye-catching and potentially due to different filters excluded genes in the hybrid validation experiment 3 part 1 on VRE data.

Figure S7 msystems.01080-24-s0007.pdf Integrative Genomics Viewer screenshot of a genomic area that shows read with variant patterns of a possible gene duplication.

Supplemental Tables msystems.01080-24-s0008.xlsx Tables S1 to S22.

ASM does not own the copyrights to Supplemental Material that may be linked to, or accessed through, an article. The authors have granted ASM a non-exclusive, world-wide license to publish the Supplemental Material files. Please contact the corresponding author directly for reuse.
