# Supplementary material for: NanoCore: core-genome-based bacterial genomic surveillance and outbreak detection in healthcare facilities from Nanopore and Illumina data
Source: mSystems. 2024 Oct 7;9(11):e01080-24. doi: 10.1128/msystems.01080-24 (PMC11575142; doi:10.1128/msystems.01080-24)
Supplement: Figure S1 — Experiment 1 (MRSA): basic statistic plots. [file msystems.01080-24-s0001.pdf]

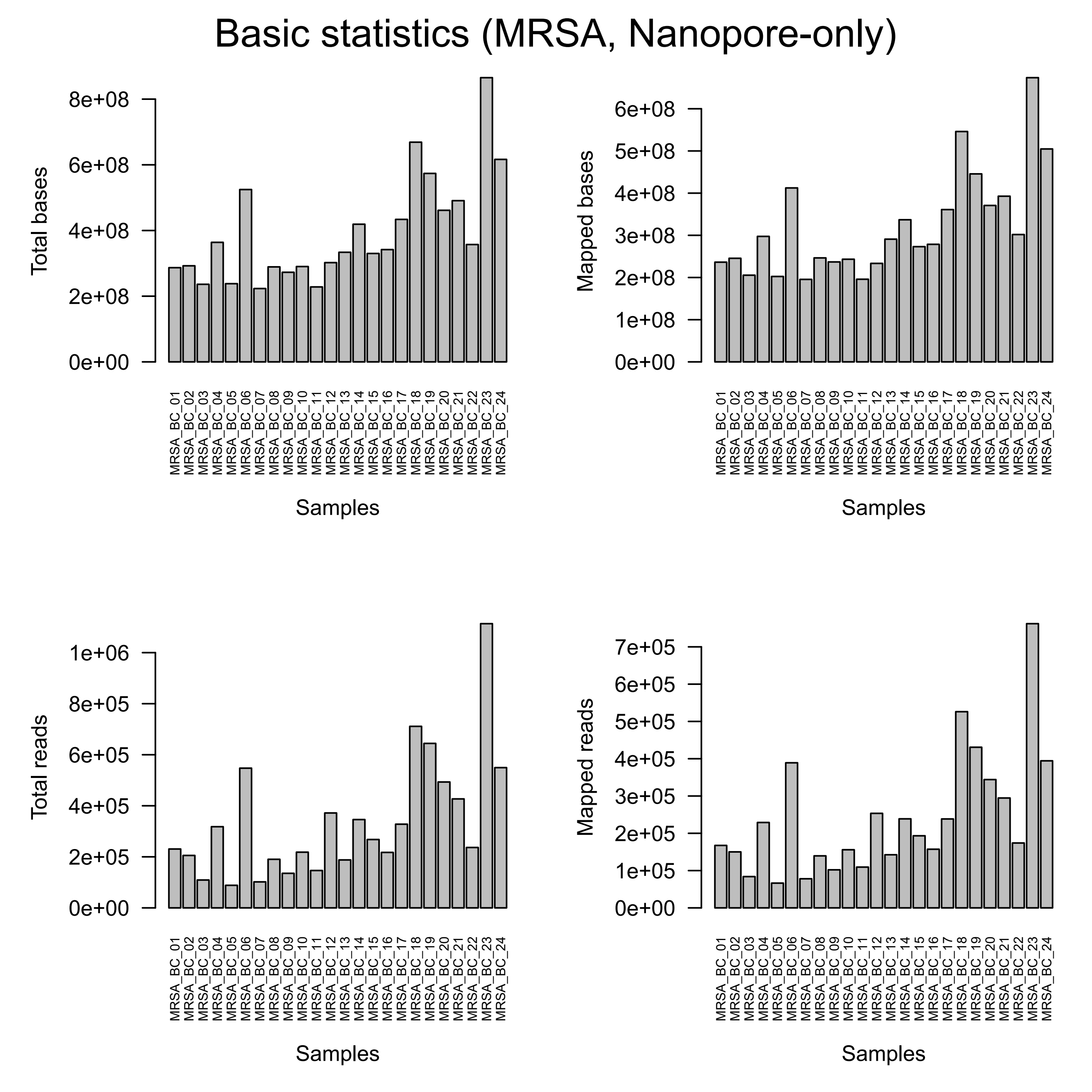
Basic statistics (MRSA, Nanopore-only)
Total bases
Mapped bases
Total reads
Mapped reads
Samples
0e+00
2e+08
4e+08
6e+08
8e+08
1e+08
3e+08
5e+08
2e+05
4e+05
6e+05
8e+05
1e+06
1e+05
3e+05
5e+05
7e+05
MRSA_BC_01
MRSA_BC_02
MRSA_BC_03
MRSA_BC_04
MRSA_BC_05
MRSA_BC_06
MRSA_BC_07
MRSA_BC_08
MRSA_BC_09
MRSA_BC_10
MRSA_BC_11
MRSA_BC_12
MRSA_BC_13
MRSA_BC_14
MRSA_BC_15
MRSA_BC_16
MRSA_BC_17
MRSA_BC_18
MRSA_BC_19
MRSA_BC_20
MRSA_BC_21
MRSA_BC_22
MRSA_BC_23
MRSA_BC_24

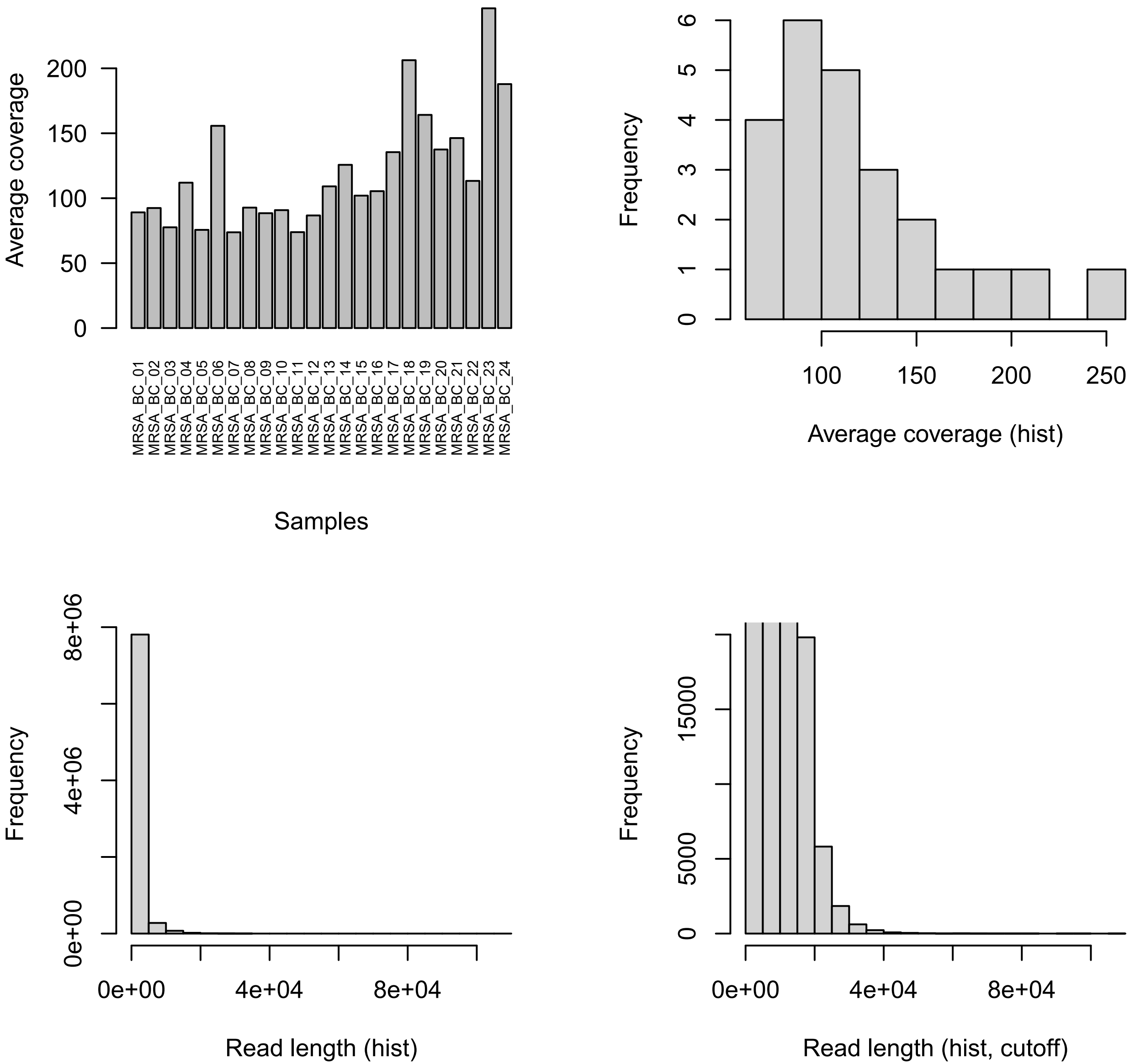

Average coverage
0
50
100
150
200
MRSA_BC_01
MRSA_BC_02
MRSA_BC_03
MRSA_BC_04
MRSA_BC_05
MRSA_BC_06
MRSA_BC_07
MRSA_BC_08
MRSA_BC_09
MRSA_BC_10
MRSA_BC_11
MRSA_BC_12
MRSA_BC_13
MRSA_BC_14
MRSA_BC_15
MRSA_BC_16
MRSA_BC_17
MRSA_BC_18
MRSA_BC_19
MRSA_BC_20
MRSA_BC_21
MRSA_BC_22
MRSA_BC_23
MRSA_BC_24
Samples
Frequency
0
1
2
3
4
5
6
100
150
200
250
Average coverage (hist)
Frequency
0e+00
4e+06
8e+06
0e+00
4e+04
8e+04
Read length (hist)
Frequency
0
5000
15000
0e+00
4e+04
8e+04
Read length (hist, cutoff)

Supplementary Figure 1: Basic statistics of the Nanopore-only validation experiment 1 on MRSA data. Shown are the number of total bases, the number of mapped bases, the number of total reads, the number of mapped reads and the average coverage, each per sample, as well as frequency histograms of the average coverage and the read length.
