# Supplementary material for: NanoCore: core-genome-based bacterial genomic surveillance and outbreak detection in healthcare facilities from Nanopore and Illumina data
Source: mSystems. 2024 Oct 7;9(11):e01080-24. doi: 10.1128/msystems.01080-24 (PMC11575142; doi:10.1128/msystems.01080-24)
Supplement: Figure S2 — Heatmaps of eye-catching and potentially due to different filters excluded genes in the Nanopore-only validation experiment 1 on MRSA data. [file msystems.01080-24-s0002.pdf]

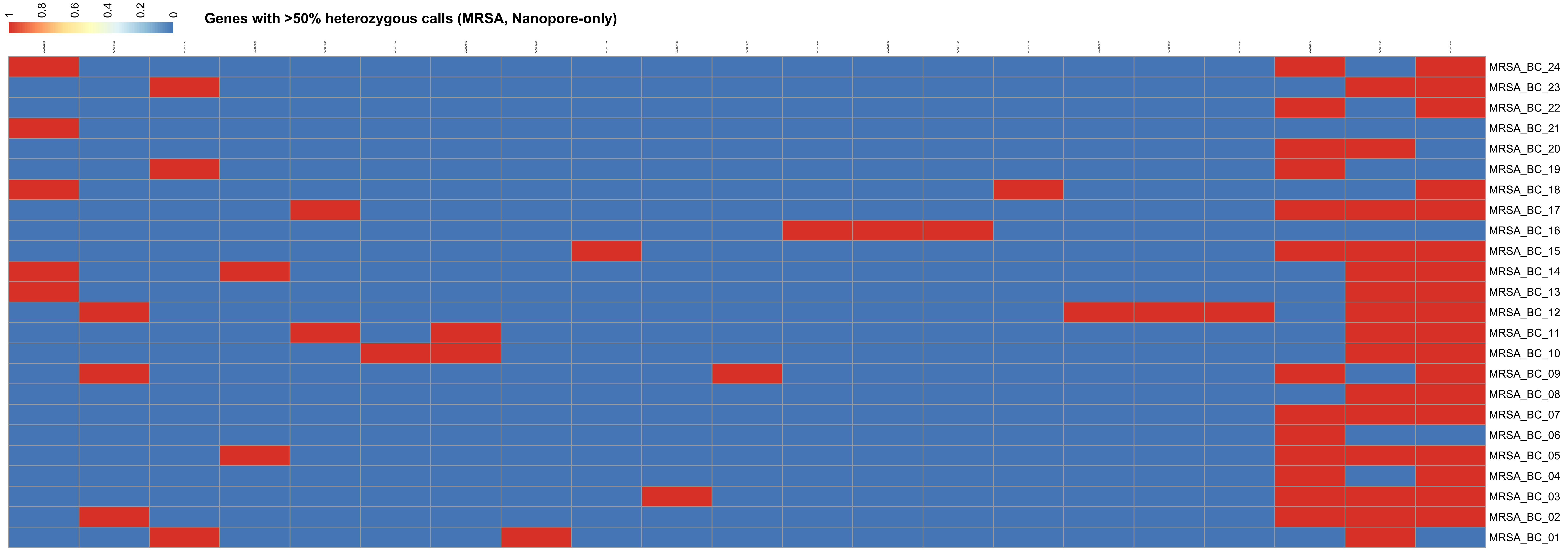
Genes with >50% heterozygous calls (MRSA, Nanopore-only)
1
0.8
0.6
0.4
0.2
0
MRSA_BC_24
MRSA_BC_23
MRSA_BC_22
MRSA_BC_21
MRSA_BC_20
MRSA_BC_19
MRSA_BC_18
MRSA_BC_17
MRSA_BC_16
MRSA_BC_15
MRSA_BC_14
MRSA_BC_13
MRSA_BC_12
MRSA_BC_11
MRSA_BC_10
MRSA_BC_09
MRSA_BC_08
MRSA_BC_07
MRSA_BC_06
MRSA_BC_05
MRSA_BC_04
MRSA_BC_03
MRSA_BC_02
MRSA_BC_01

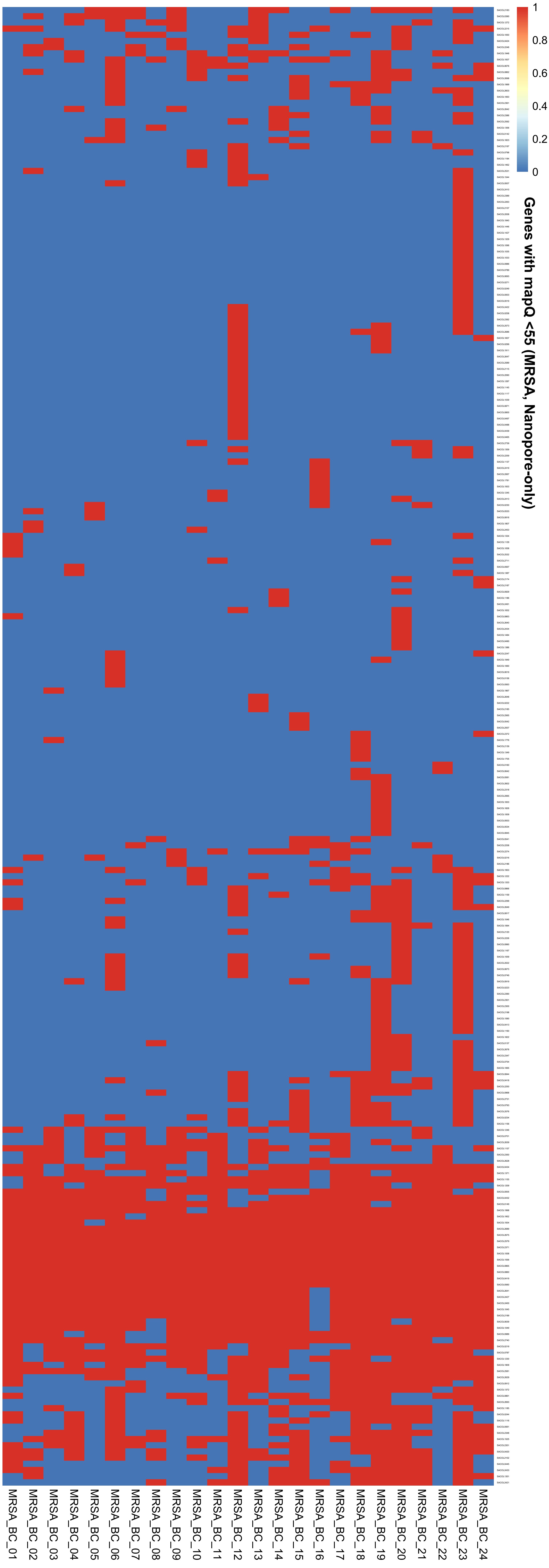
Genes with mapQ <55 (MRSA, Nanopore-only)
1
0.8
0.6
0.4
0.2
0
MRSA_BC_01
MRSA_BC_02
MRSA_BC_03
MRSA_BC_04
MRSA_BC_05
MRSA_BC_06
MRSA_BC_07
MRSA_BC_08
MRSA_BC_09
MRSA_BC_10
MRSA_BC_11
MRSA_BC_12
MRSA_BC_13
MRSA_BC_14
MRSA_BC_15
MRSA_BC_16
MRSA_BC_17
MRSA_BC_18
MRSA_BC_19
MRSA_BC_20
MRSA_BC_21
MRSA_BC_22
MRSA_BC_23
MRSA_BC_24

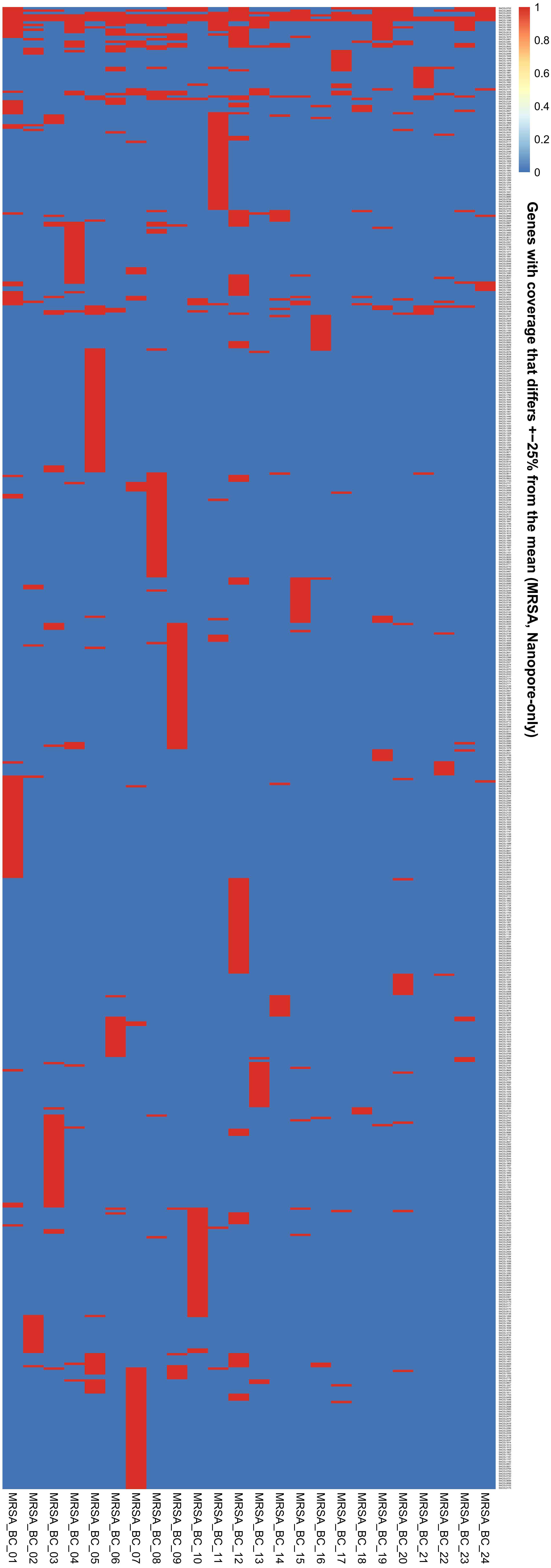

Genes with coverage that differs +-25% from the mean (MRSA, Nanopore-only)
1
0.8
0.6
0.4
0.2
0
MRSA_BC_01
MRSA_BC_02
MRSA_BC_03
MRSA_BC_04
MRSA_BC_05
MRSA_BC_06
MRSA_BC_07
MRSA_BC_08
MRSA_BC_09
MRSA_BC_10
MRSA_BC_11
MRSA_BC_12
MRSA_BC_13
MRSA_BC_14
MRSA_BC_15
MRSA_BC_16
MRSA_BC_17
MRSA_BC_18
MRSA_BC_19
MRSA_BC_20
MRSA_BC_21
MRSA_BC_22
MRSA_BC_23
MRSA_BC_24

Supplementary Figure 2: Heatmaps of eye-catching and potentially due to different filters excluded genes in the Nanopore-only validation experiment 1 on MRSA data. Shown are genes with more than 50% of heterozygous variant-calls, genes with below 55 mapping quality and genes with a coverage that differs more than 25% from the mean.
