# Supplementary material for: NanoCore: core-genome-based bacterial genomic surveillance and outbreak detection in healthcare facilities from Nanopore and Illumina data
Source: mSystems. 2024 Oct 7;9(11):e01080-24. doi: 10.1128/msystems.01080-24 (PMC11575142; doi:10.1128/msystems.01080-24)
Supplement: Figure S3 — Experiment 2 (VRE): basic statistic plots. [file msystems.01080-24-s0003.pdf]

Basic statistics (VRE, Nanopore-only)

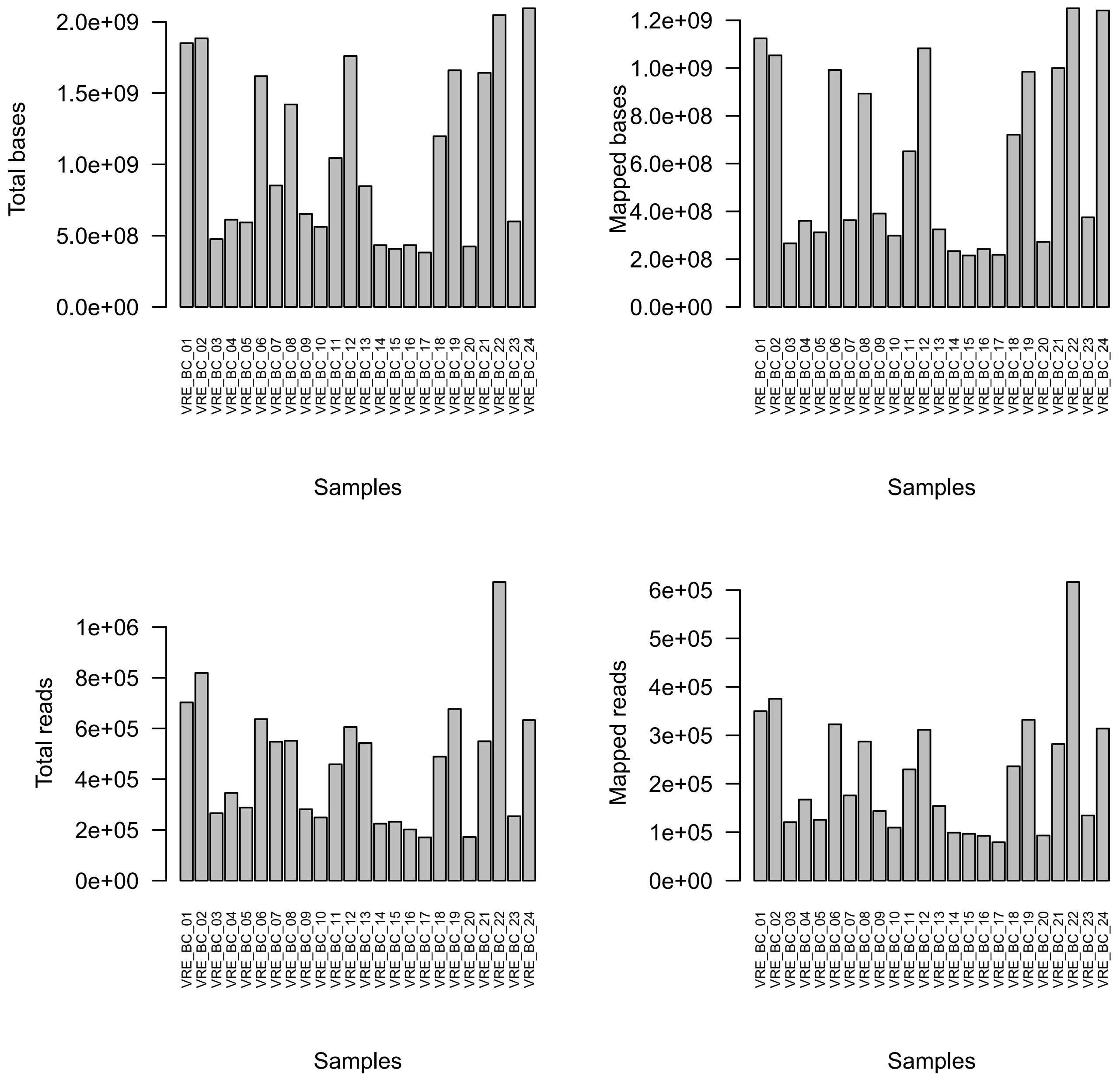

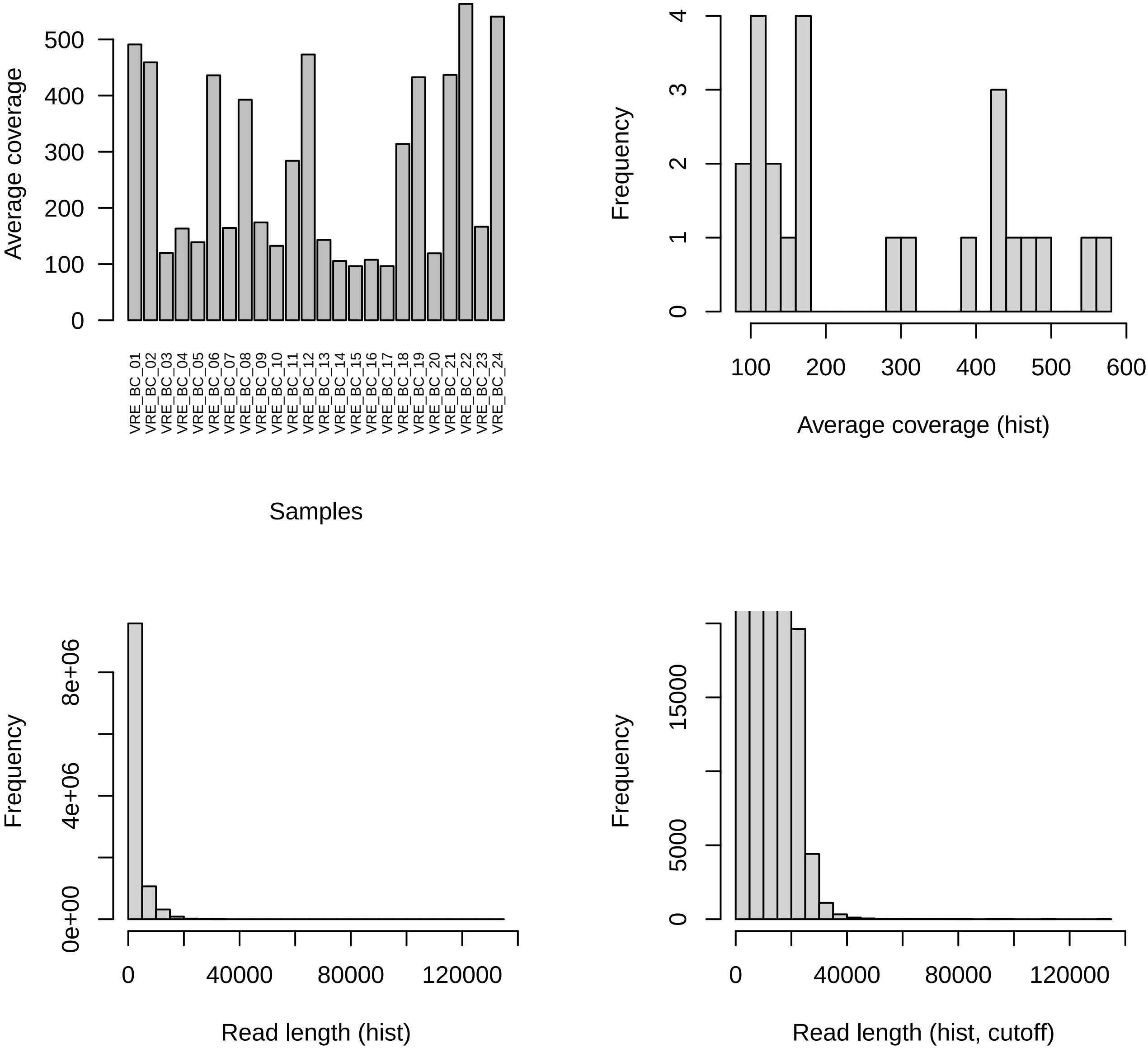
Average coverage
Samples
VRE_BC_01
VRE_BC_02
VRE_BC_03
VRE_BC_04
VRE_BC_05
VRE_BC_06
VRE_BC_07
VRE_BC_08
VRE_BC_09
VRE_BC_10
VRE_BC_11
VRE_BC_12
VRE_BC_13
VRE_BC_14
VRE_BC_15
VRE_BC_16
VRE_BC_17
VRE_BC_18
VRE_BC_19
VRE_BC_20
VRE_BC_21
VRE_BC_22
VRE_BC_23
VRE_BC_24
Frequency
Average coverage (hist)
Frequency
Read length (hist)
Frequency
Read length (hist, cutoff)

Supplementary Figure 3: Basic statistics of the Nanopore-only validation experiment 2 on VRE data. Shown are the number of total bases, the number of mapped bases, the number of total reads, the number of mapped reads and the average coverage, each per sample, as well as frequency histograms of the average coverage and the read length.
