# Supplementary material for: NanoCore: core-genome-based bacterial genomic surveillance and outbreak detection in healthcare facilities from Nanopore and Illumina data
Source: mSystems. 2024 Oct 7;9(11):e01080-24. doi: 10.1128/msystems.01080-24 (PMC11575142; doi:10.1128/msystems.01080-24)
Supplement: Figure S4 — Heatmaps of eye-catching and potentially due to different filters excluded genes in the Nanopore-only validation experiment 2 on VRE data. [file msystems.01080-24-s0004.pdf]

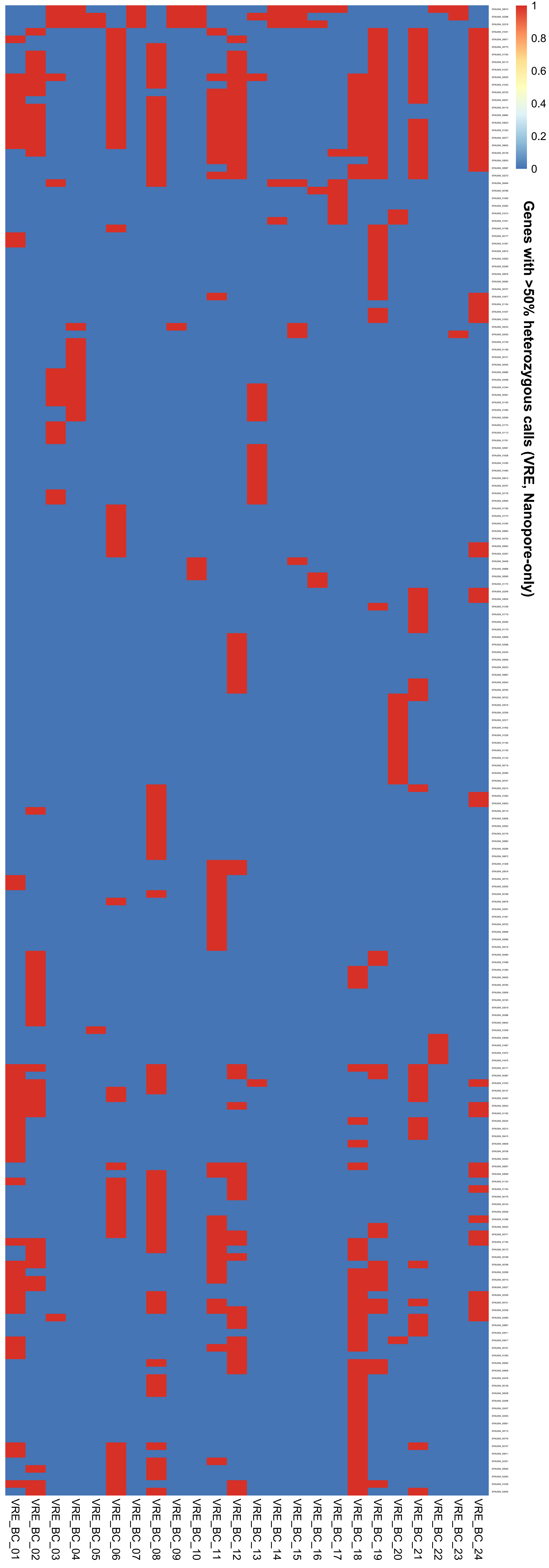
Genes with >50% heterozygous calls (VRE, Nanopore-only)
1
0.8
0.6
0.4
0.2
0
VRE_BC_01
VRE_BC_02
VRE_BC_03
VRE_BC_04
VRE_BC_05
VRE_BC_06
VRE_BC_07
VRE_BC_08
VRE_BC_09
VRE_BC_10
VRE_BC_11
VRE_BC_12
VRE_BC_13
VRE_BC_14
VRE_BC_15
VRE_BC_16
VRE_BC_17
VRE_BC_18
VRE_BC_19
VRE_BC_20
VRE_BC_21
VRE_BC_22
VRE_BC_23
VRE_BC_24

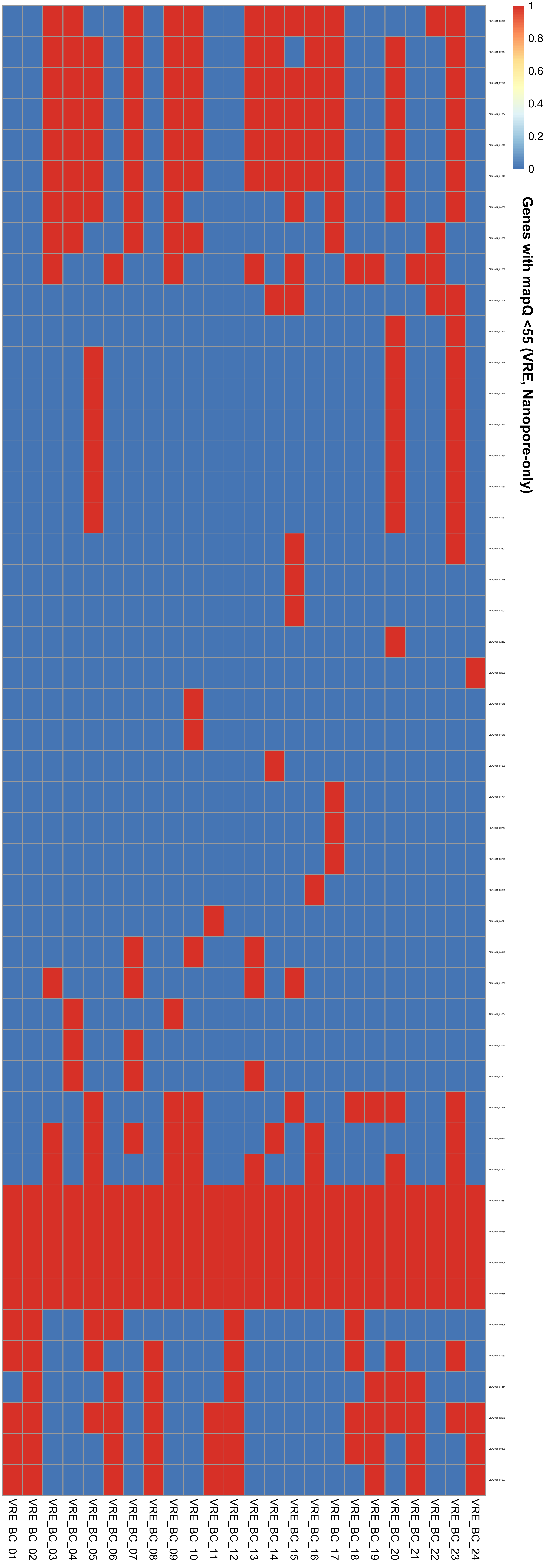

Genes with mapQ <55 (VRE, Nanopore-only)
1
0.8
0.6
0.4
0.2
0
VRE_BC_01
VRE_BC_02
VRE_BC_03
VRE_BC_04
VRE_BC_05
VRE_BC_06
VRE_BC_07
VRE_BC_08
VRE_BC_09
VRE_BC_10
VRE_BC_11
VRE_BC_12
VRE_BC_13
VRE_BC_14
VRE_BC_15
VRE_BC_16
VRE_BC_17
VRE_BC_18
VRE_BC_19
VRE_BC_20
VRE_BC_21
VRE_BC_22
VRE_BC_23
VRE_BC_24

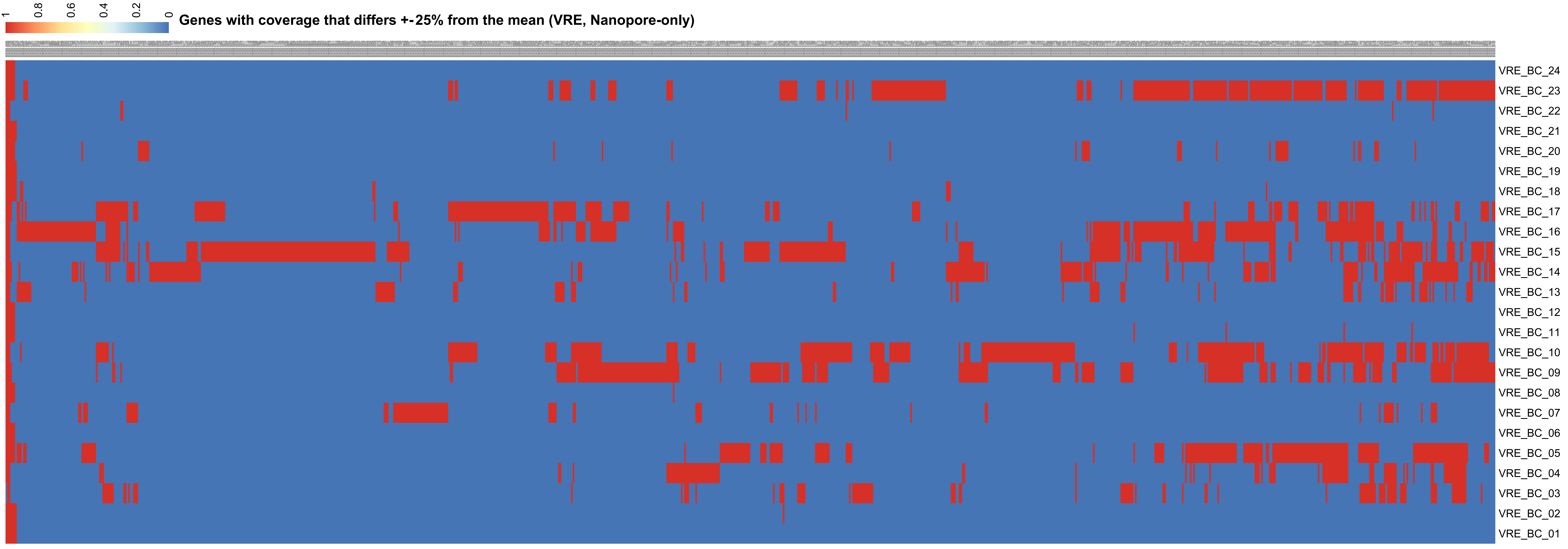
Genes with coverage that differs +-25% from the mean (VRE, Nanopore-only)
1
0.8
0.6
0.4
0.2
0
VRE_BC_24
VRE_BC_23
VRE_BC_22
VRE_BC_21
VRE_BC_20
VRE_BC_19
VRE_BC_18
VRE_BC_17
VRE_BC_16
VRE_BC_15
VRE_BC_14
VRE_BC_13
VRE_BC_12
VRE_BC_11
VRE_BC_10
VRE_BC_09
VRE_BC_08
VRE_BC_07
VRE_BC_06
VRE_BC_05
VRE_BC_04
VRE_BC_03
VRE_BC_02
VRE_BC_01

Supplementary Figure 4: Heatmaps of eye-catching and potentially due to different filters excluded genes in the Nanopore-only validation experiment 2 on VRE data. Shown are genes with more than 50% of heterozygous variant-calls, genes with below 55 mapping quality and genes with a coverage that differs more than 25% from the mean.
