# Supplementary material for: NanoCore: core-genome-based bacterial genomic surveillance and outbreak detection in healthcare facilities from Nanopore and Illumina data
Source: mSystems. 2024 Oct 7;9(11):e01080-24. doi: 10.1128/msystems.01080-24 (PMC11575142; doi:10.1128/msystems.01080-24)
Supplement: Figure S6 — Heatmaps of eye-catching and potentially due to different filters excluded genes in the hybrid validation experiment 3 part 1 on VRE data. [file msystems.01080-24-s0006.pdf]

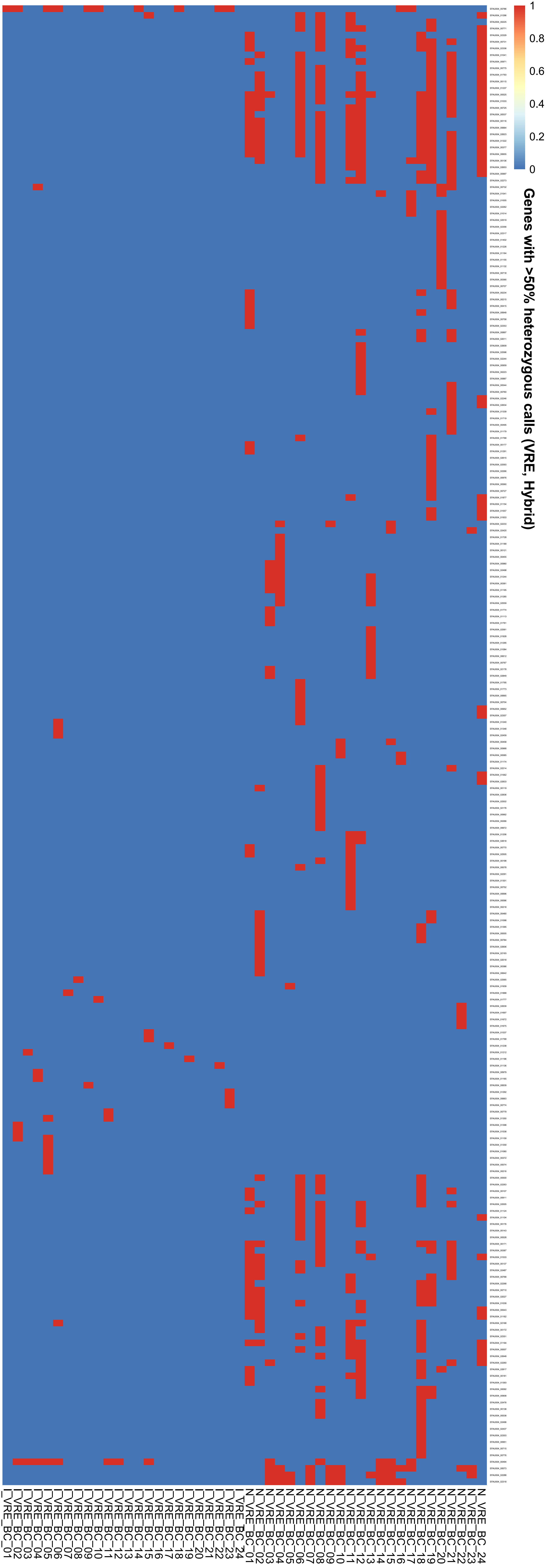
Genes with >50% heterozygous calls (VRE, Hybrid)
1
0.8
0.6
0.4
0.2
0
I_VRE_BC_01
I_VRE_BC_02
I_VRE_BC_03
I_VRE_BC_04
I_VRE_BC_05
I_VRE_BC_06
I_VRE_BC_07
I_VRE_BC_08
I_VRE_BC_09
I_VRE_BC_10
I_VRE_BC_11
I_VRE_BC_12
I_VRE_BC_13
I_VRE_BC_14
I_VRE_BC_15
I_VRE_BC_16
I_VRE_BC_17
I_VRE_BC_18
I_VRE_BC_19
I_VRE_BC_20
I_VRE_BC_21
I_VRE_BC_22
I_VRE_BC_23
I_VRE_BC_24
N_VRE_BC_01
N_VRE_BC_02
N_VRE_BC_03
N_VRE_BC_04
N_VRE_BC_05
N_VRE_BC_06
N_VRE_BC_07
N_VRE_BC_08
N_VRE_BC_09
N_VRE_BC_10
N_VRE_BC_11
N_VRE_BC_12
N_VRE_BC_13
N_VRE_BC_14
N_VRE_BC_15
N_VRE_BC_16
N_VRE_BC_17
N_VRE_BC_18
N_VRE_BC_19
N_VRE_BC_20
N_VRE_BC_21
N_VRE_BC_22
N_VRE_BC_23
N_VRE_BC_24

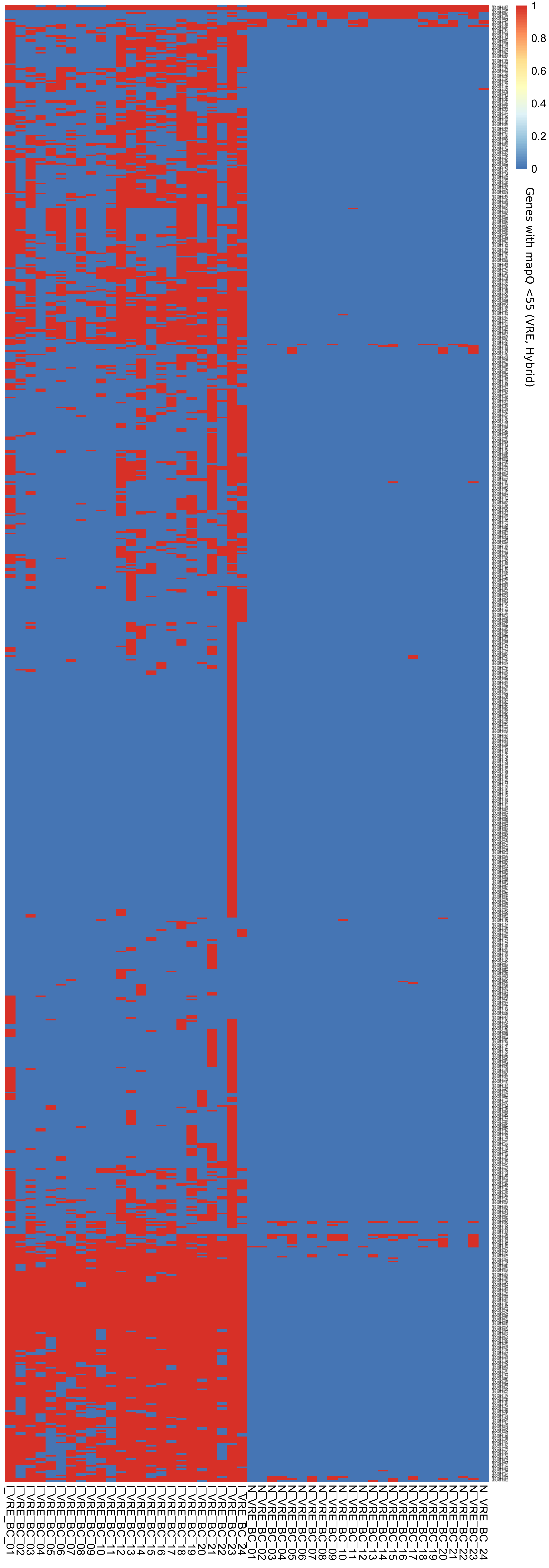

1
0.8
0.6
0.4
0.2
0
Genes with mapQ <55 (VRE, Hybrid)
I_VRE_BC_01
I_VRE_BC_02
I_VRE_BC_03
I_VRE_BC_04
I_VRE_BC_05
I_VRE_BC_06
I_VRE_BC_07
I_VRE_BC_08
I_VRE_BC_09
I_VRE_BC_10
I_VRE_BC_11
I_VRE_BC_12
I_VRE_BC_13
I_VRE_BC_14
I_VRE_BC_15
I_VRE_BC_16
I_VRE_BC_17
I_VRE_BC_18
I_VRE_BC_19
I_VRE_BC_20
I_VRE_BC_21
I_VRE_BC_22
I_VRE_BC_23
I_VRE_BC_24
N_VRE_BC_01
N_VRE_BC_02
N_VRE_BC_03
N_VRE_BC_04
N_VRE_BC_05
N_VRE_BC_06
N_VRE_BC_07
N_VRE_BC_08
N_VRE_BC_09
N_VRE_BC_10
N_VRE_BC_11
N_VRE_BC_12
N_VRE_BC_13
N_VRE_BC_14
N_VRE_BC_15
N_VRE_BC_16
N_VRE_BC_17
N_VRE_BC_18
N_VRE_BC_19
N_VRE_BC_20
N_VRE_BC_21
N_VRE_BC_22
N_VRE_BC_23
N_VRE_BC_24

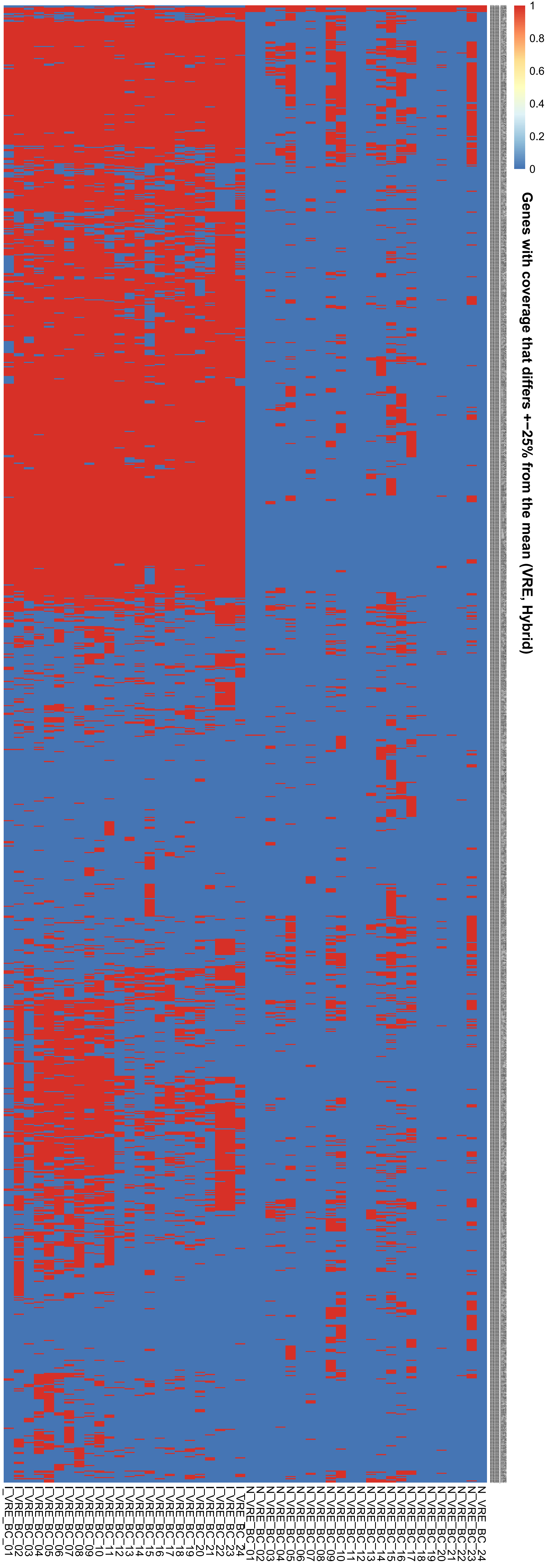
1
0.8
0.6
0.4
0.2
0
Genes with coverage that differs +−25% from the mean (VRE, Hybrid)
I_VRE_BC_01
I_VRE_BC_02
I_VRE_BC_03
I_VRE_BC_04
I_VRE_BC_05
I_VRE_BC_06
I_VRE_BC_07
I_VRE_BC_08
I_VRE_BC_09
I_VRE_BC_10
I_VRE_BC_11
I_VRE_BC_12
I_VRE_BC_13
I_VRE_BC_14
I_VRE_BC_15
I_VRE_BC_16
I_VRE_BC_17
I_VRE_BC_18
I_VRE_BC_19
I_VRE_BC_20
I_VRE_BC_21
I_VRE_BC_22
I_VRE_BC_23
I_VRE_BC_24
N_VRE_BC_01
N_VRE_BC_02
N_VRE_BC_03
N_VRE_BC_04
N_VRE_BC_05
N_VRE_BC_06
N_VRE_BC_07
N_VRE_BC_08
N_VRE_BC_09
N_VRE_BC_10
N_VRE_BC_11
N_VRE_BC_12
N_VRE_BC_13
N_VRE_BC_14
N_VRE_BC_15
N_VRE_BC_16
N_VRE_BC_17
N_VRE_BC_18
N_VRE_BC_19
N_VRE_BC_20
N_VRE_BC_21
N_VRE_BC_22
N_VRE_BC_23
N_VRE_BC_24

Supplementary Figure 6: Heatmaps of eye-catching and potentially due to different filters excluded genes in the hybrid validation experiment 3 part 1 on VRE data. Shown are genes with more than 50% of heterozygous variant-calls, genes with below 55 mapping quality and genes with a coverage that differs more than 25% from the mean.
