# Supplementary material for: NanoCore: core-genome-based bacterial genomic surveillance and outbreak detection in healthcare facilities from Nanopore and Illumina data
Source: mSystems. 2024 Oct 7;9(11):e01080-24. doi: 10.1128/msystems.01080-24 (PMC11575142; doi:10.1128/msystems.01080-24)
Supplement: Figure S7 — Integrative Genomics Viewer screenshot of a genomic area that shows read with variant patterns of a possible gene duplication. [file msystems.01080-24-s0007.pdf]

IGV screenshots of possible gene duplication

A

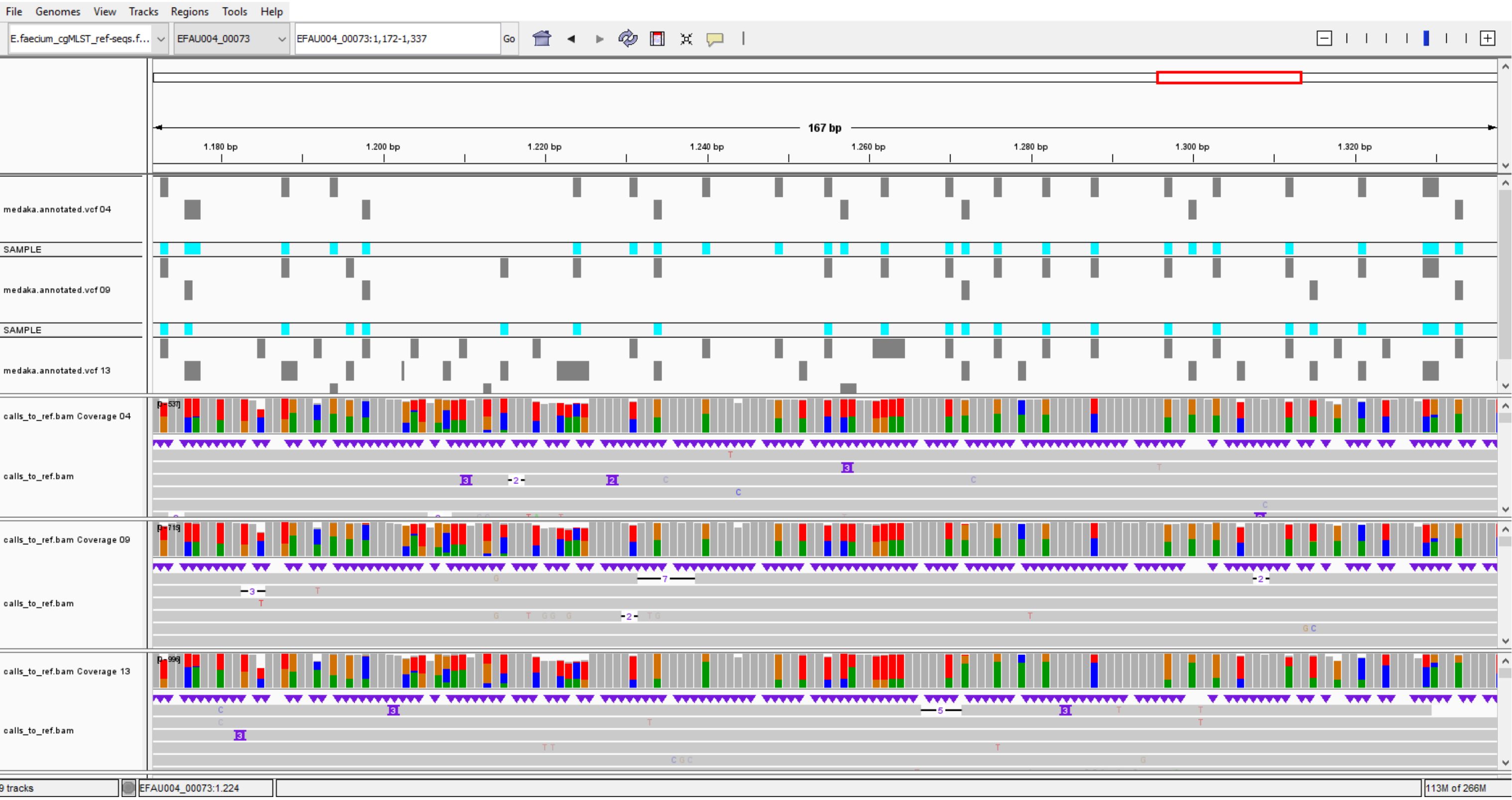

B

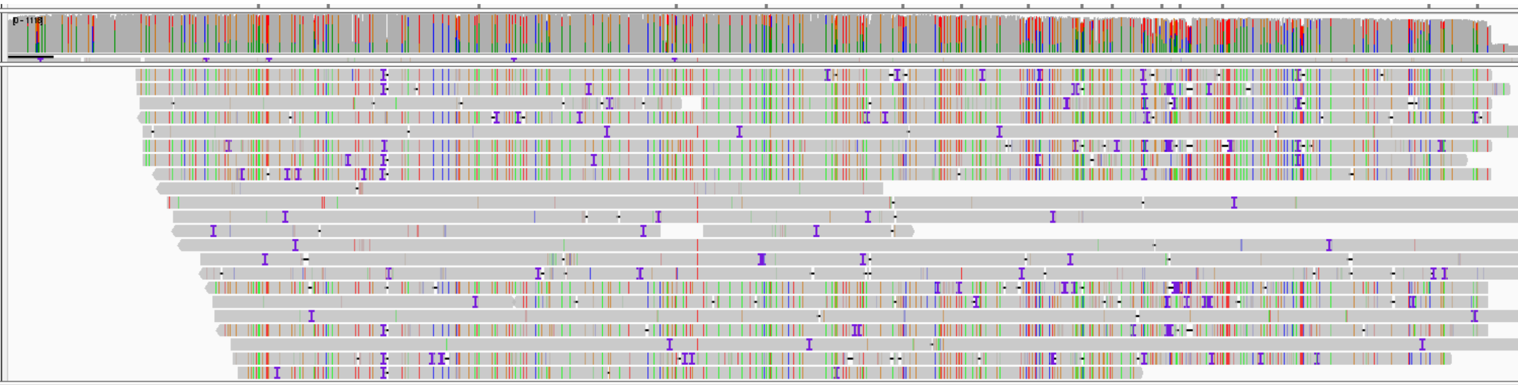

Supplementary Figure 7: Integrative Genomics Viewer screenshot of a genomic area that shows read with variant patterns of a possible gene duplication. Shown are A) a zoomed-in position of gene EFAU004_00073 that shows that most of the found variants indicate two different bases with around 50% ratio each and B) the full length of gene EFAU004_00073 that show, that all reads exhibit one of two possible variant patterns.
